# Epidemiology and healthcare burden of non-fatal maxillofacial injuries in Bangladesh: Evidence from Bangladesh Health and Injury Survey (BHIS), 2016

**DOI:** 10.1371/journal.pone.0353183

**Published:** 2026-07-09

**Authors:** Ruksana Sharmin Swarna, Mariam Zamila, Imteaz Mahmud, Israt Jahan Bushra, Monjur Rahman, Salim Mahmud Chowdhury, Mohammad Delwer Hossain Hawlader, Saidur Rahman Mashreky

**Affiliations:** 1 Department of Public Health, School of Health and Life Sciences, North South University, Bashundhara, Dhaka, Bangladesh; 2 Centre for Injury Prevention and Research, Bangladesh (CIPRB), Mohakhali, Dhaka, Bangladesh; University of the Witwatersrand Johannesburg Faculty of Health Sciences, SOUTH AFRICA

## Abstract

**Background:**

Maxillofacial injuries cause significant morbidity and contribute greatly to the global healthcare burden, yet epidemiological estimates from Bangladesh are lacking. This is the first population-level study to provide nationally representative estimates of the epidemiology, healthcare burden, and economic impact of such injuries in Bangladesh.

**Methods:**

A secondary analysis was conducted using data from the Bangladesh Health and Injury Survey 2016, a national cross-sectional survey that utilised multistage cluster sampling to collect data from 292,217 individuals across all age groups with a six-month recall period. Annualised incidence rates (IRs) and incidence rate ratios (IRRs), with 95% confidence intervals (CIs), were estimated and stratified by sociodemographic, injury, and healthcare-related characteristics.

**Results:**

The annual IR of non-fatal maxillofacial injuries was 793 (95% CI 760.3–824.8) per 100,000 population in 2016. Males (IR 989.1 per 100,000; 95% CI 938.1–1040.2), children aged 0–4 years (IR 1577.9 per 100,000; 95% CI 1414.9–1740.9), urban residents (IR 826.1 per 100,000; 95% CI 748.1–904.2), and auto-rickshaw/bus/truck drivers (IR 1632.7 per 100,000; 95% CI 1205.0–2060.3) experienced higher rates. Falls and road traffic accidents accounted for most cases, and 56.9% of injured individuals sustained concomitant injuries. Moreover, 47.4% of the injured did not receive any treatment, while 15.1% required hospital admission. The mean inpatient out-of-pocket (OOP) expenditure was USD 470.5 [standard deviation (SD) 1497.1]. Extrapolating the BHIS 2016 estimates to the 2026 national population projected an increase in non-fatal maxillofacial injury cases from 1.27 to 1.41 million.

**Conclusion:**

A high incidence rate, a substantial treatment gap, and significant OOP costs underscore critical gaps in maxillofacial injury care in Bangladesh. Alongside reinforcing the country’s infrastructure, we call for targeted policy reforms to strengthen injury prevention, treatment, rehabilitation, and financial protection.

## Introduction

The human face is a complex organ comprising distinct functional and aesthetic subunits, each with different yet interconnected anatomical and physiological roles [1]. Injuries in this region can result in profound physical, functional, and psychological impairments, often imposing considerable economic burdens through high treatment costs, prolonged hospitalisations, and lost productivity [2–5]. Blunt or penetrating injuries can be confined solely to soft tissues or associated with underlying single or multiple craniofacial fractures [6]. Clinical presentations may include bleeding, ecchymosis, oedema, subconjunctival haemorrhage, crepitus, step deformity, mobility, and altered sensory or motor functions [7–10]. Prior research found that 16% to 70% of maxillofacial injury cases sustained concomitant injuries to other body parts [11–14]. Though many cases can be minor, severe forms can threaten life by damaging nearby vital structures, such as the brain and the airway [7,15]. Hence, management may require a multidisciplinary approach to optimise patients’ prognosis, following emergency airway maintenance, haemorrhage control, and hemodynamic stabilisation [7,16]. Importantly, early childhood trauma can also potentially disrupt the normal growth of facial bones and soft tissues, leading to functional impairment and psychological distress caused by visible disfigurement, creating a dual burden later in life [4,17–19].

The high incidence of maxillofacial injuries imposes significant demands on health systems worldwide [20]. According to the National Trauma Data Bank report 2016 from the United States, nearly 25% of all injuries involved the face [15]. The Global Burden of Disease (GBD) 2019 study reported that facial fractures alone accounted for approximately 10.7 million incident cases, representing nearly one-seventh of all bone fractures worldwide—a 19.4% increase from 1990 [2]. Review studies from sub-Saharan Africa and many other low- and middle-income countries (LMICs) revealed that adults predominantly sustained soft tissue injuries, while the mandible was the most common bone to be fractured [21,22]. At the emergency department of a tertiary care hospital in eastern Nepal, isolated soft tissue injuries were reported to account for 48.7% of all maxillofacial trauma cases, with lacerations being the most frequent type [23].

Worldwide, numerous single- and multi-centre hospital-based studies concentrated on surgically and non-surgically managed cases of maxillofacial fractures, and Bangladesh is no exception [7,24–27]. Some studies reported associated soft tissue injuries [21–23,28,29]. The predominant causes include falls, road traffic accidents (RTAs), interpersonal violence, sports, occupational hazards, and human or animal bites [2,22,27,30–32], although their relative rankings vary across socioeconomic and geographic contexts globally [33]. A single-centre study in Bangladesh reported that 81% of the maxillofacial fractures were caused by RTAs [27]. Most craniomaxillofacial injuries were reported to stem from RTAs across many other LMICs [22,27,34,35]. Despite the alarming prevalence of RTAs, there remains a paucity of epidemiological and healthcare-related data on the nature and characteristics of the full spectrum of maxillofacial injuries from a population perspective in these regions [36,37].

Bangladesh Health and Injury Survey (BHIS) is one of the largest nationwide injury studies conducted in LMIC settings, providing insights into the epidemiology and healthcare utilisation of diverse injuries. The first BHIS in 2003 focused on injury patterns among children under 18 years of age and offered limited insight into the burden and epidemiology of injuries in adults [38,39]. Another national study reported overall fatal and non-fatal injury outcomes among rural populations in 2013, and maxillofacial injuries were not presented as a distinct category [40]. As a consequence, a major gap remains in understanding the epidemiology of maxillofacial injuries across all age groups in urban and rural Bangladesh. Notably, the second BHIS in 2016 was robustly designed to address the lack of more inclusively detailed population-level estimates to inform the burden and characteristics of fatal and non-fatal injuries in the country [41]. The present study aimed to delineate the epidemiology and healthcare burden of non-fatal maxillofacial injuries across all sociodemographic groups in Bangladesh through a secondary analysis of nationally representative data from the BHIS 2016, filling a crucial knowledge gap in injury research within LMIC contexts. The analysis captured the complete spectrum of both treated and untreated non-fatal maxillofacial injury cases across all ages, healthcare utilisation categories, severity levels, geographic strata, and associated out-of-pocket costs. Overall, the findings generate large-scale and improved data-driven evidence to inform infrastructure advancement and health policy planning, especially to strengthen injury prevention strategies, trauma care, and rehabilitation services in Bangladesh and other LMICs.

## Methods

### Study design and setting

The current study was based on data from the BHIS 2016, conducted in Bangladesh. The BHIS 2016 was a large-scale, community-based cross-sectional survey conducted between March and June in that year. This nationally representative survey followed a multistage cluster sampling design utilising probability-proportional-to-size (PPS) methodology and incorporated both discrete urban and rural sampling units. Data for this survey were collected using a questionnaire covering the following six modules: (1) household and socioeconomic information, (2) verbal autopsy, (3) injury morbidity, (4) injury mortality, (5) injury mechanism, and (6) quality-of-life assessment. All these modules are presented in the S1 File. In this study, a secondary analysis was carried out using the morbidity module, which contained information based on a six-month recall period to capture only non-fatal injury events. The study also followed the Strengthening the Reporting of Observational Studies in Epidemiology (STROBE) guidelines to report the findings [42].

### Sampling strategy and data collection

**Fig 1:**
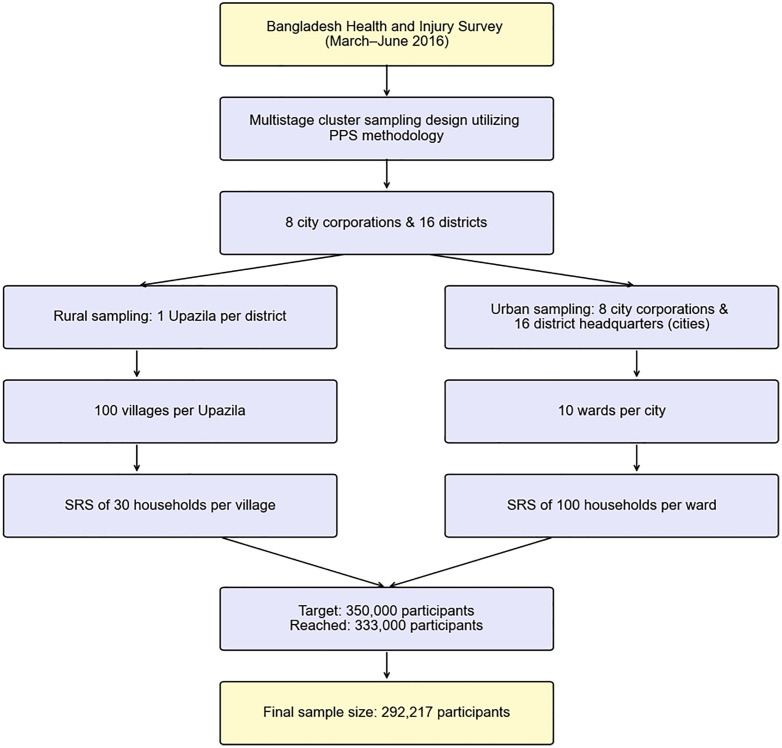
Sampling and participant selection. Flow diagram illustrating the sampling process and study participant selection for the final analysis; Bangladesh Health and Injury Survey, 2016. PPS = probability-proportional-to-size.

In this survey, 16 districts were randomly selected from the 64 districts across the 8 divisions (clusters) of Bangladesh. For rural areas, one Upazila was randomly chosen from each district (cluster). Following this, 100 villages were randomly selected from each Upazila (cluster), and each village comprised approximately 300 households. Subsequently, 30 households were selected from each village using systematic random sampling (SRS). The urban sample was drawn from 8 randomly selected city corporations (clusters) and previously selected 16 district headquarters (clusters). Following this, 10 wards were randomly selected from each city corporation and district headquarters. After that, the SRS technique was applied to select 100 households from each ward. For data collection, 64 formally trained data collectors and 16 experienced supervisors were employed. All personnel were field staff from the Centre for Injury Prevention and Research, Bangladesh (CIPRB), with experience in previous household surveys. For face-to-face interviews, household heads were considered the primary respondents for all adult household members. Mothers were considered the primary respondents for their children (aged ≤12 years), whereas caregivers were interviewed otherwise. Whenever it was not possible to contact the preferred household member, the most knowledgeable member present at the time of the interview was considered the primary respondent. A household member was defined as a person who lived in the same dwelling and shared meals with the family, including domestic helpers and long-term guests [41]. For this study, an injury case was operationally defined as any instance in which a household member sustained a non-fatal maxillofacial injury and either sought treatment or lost at least 1 day of work, school, or the ability to perform usual activities [41,43]. Primary respondents were asked whether each household member had experienced a new injury event (i.e., an incident case) during the preceding six months. To confirm the incident nature of a reported case within this predefined recall period, data collectors also encouraged other household members to verify the case and associated information to minimise recall bias [41]. Since the survey was not restricted to first injury events, primary respondents were asked to report all non-fatal maxillofacial injury events meeting the operational case definition that occurred within the recall window, and each qualifying event was duly recorded. Critically, case identification was anchored to the temporal onset of the injury event rather than to the affected person’s current injury status at the time of the interview. Injury events with onsets predating the six-month recall window were not counted, regardless of whether the affected individual was still receiving treatment or experienced any ongoing functional limitation within the six-month recall window or even at the time of data collection. If an eligible non-fatal injury was identified, the interviewer administered additional questionnaires to primary respondents according to the data-entry algorithms. All data were collected using tablets equipped with REDCap (Research Electronic Data Capture) software [44]. Field data were transferred to a central server and downloaded thereafter for cleaning and analysis. Overall, 350,000 people were targeted for sampling, and 333,000 were successfully reached. The details of the sample size calculation are provided in the S2 File. Data from 292,217 participants were included in the final analysis following cleaning and validation (Fig 1).

### Data source and extraction

This study utilised secondary data from the Bangladesh Health and Injury Survey (BHIS) 2016, which received ethical approval from the Ethics Review Committee (ERC) of CIPRB (reference number: CIPRB/ERC/2016/005). The dataset was requested and obtained from CIPRB with institutional permission. After reviewing the dataset structure, variables related to non-fatal maxillofacial injuries and relevant independent factors were identified and extracted for analysis. As per institutional guidelines, no additional ethical approval was required for this secondary analysis. All procedures adhered to the ERC’s regulations and the principles of the Declaration of Helsinki. The BHIS 2016 confirmed that informed consent was obtained from all participants in accordance with the requirements of the ERC.

#### Patient and public involvement statement.

Patients or the public were not involved in the study design, conduct, reporting, or distribution strategies of the research.

### Study variables

The primary outcome was encountering a new case of non-fatal maxillofacial injury (incident case) within the preceding six months*.* Sociodemographic variables included sex, age, residence (urban/rural), and occupation. Injury-related variables were places of injury occurrence, injury aetiology, consciousness status after the injury event, ambulatory status after the injury event, injury severity, and concomitantly injured body regions. Healthcare-related factors included hospital treatment availability, hospital admission status, type of admitting health facility, surgery status, type of anaesthesia used, out-of-pocket (OOP) costs for inpatients, length of stay (LOS) in hospital, and days of work loss.

### Statistical analysis

Data analyses were conducted using Stata version 14. Presented case counts derived from participants’ six-month recall data. Descriptive statistics were performed to summarize sociodemographic, injury, and healthcare-related characteristics. Categorical variables were presented as frequencies and percentages. Continuous variables were examined for distributional shape using histograms and quantile–quantile (Q–Q) plots, and results were presented as mean with standard deviation (SD) or median with interquartile range (IQR), as appropriate. As BHIS used a six-month recall period to quantify the burden of non-fatal maxillofacial injuries in 2016, incidence rates (IRs) were multiplied by two to generate annual estimates and were presented as new cases per 100,000 population per year. Incidence rate ratios (IRRs) were also reported. 95% confidence intervals (CIs) for IRs and IRRs were considered. The annual burden for 2026 was also projected by extrapolating the 2016 BHIS data to the estimated national population [45].

## Results

Table 1 demonstrates the sociodemographic characteristics of survey participants. The BHIS 2016 recorded 1158 maxillofacial injury cases over a six-month recall period. There was almost an equal distribution between males (49.9%) and females (50.1%). Most individuals fell into the 25–39 years (27%) age group. Most participants resided in rural areas (64.4%). The participants’ occupations were primarily housewives (29.2%) and students (26%). Other major occupations included businessmen and day labourers.

**Table 1:** Sociodemographic characteristics of study participants.

Characteristics	Frequency (n)	Percentage (%)
**Sex**		
Female	146,431	50.1
Male	145,786	49.9
**Age (years)**		
0–4	22,814	7.81
5–9	28,130	9.69
10–17	48,622	16.6
18–24	36,626	12.5
25–39	78,815	27.0
40–59	56,445	19.3
60+	20,765	7.10
**Residence**		
Rural	188,119	64.4
Urban	104,098	35.6
**Occupation**		
Van/rickshaw driver*	3,561	1.24
Auto-rickshaw/bus/truck driver^†^	3,430	1.17
Farmer	20,639	7.08
Businessman	24,758	8.47
Construction worker	21,401	7.34
Service holder	16,807	5.75
Student	76,043	26.0
Housewife	85,400	29.2
Pre-school child	23,822	8.15
Other^‡^	16,356	5.60

Sociodemographic profile of study participants (n = 292,217) included in the analysis.

* Van and rickshaw are non-motorised three-wheeler vehicles.

† Auto-rickshaws are motorised three-wheeler vehicles.

‡ Other included mostly unemployed older adults, as well as daily wage labourers, small vendors, and domestic workers.

Table 2 presents the IRs and IRRs of maxillofacial injury-related characteristics. The overall IR in 2016 was 792.6 (95% CI 760.3–824.8) per 100,000 persons per year. It was 989.1 (95% CI 938.1–1040.2) per 100,000 per year among males, yielding a 1.66 (95% CI 1.45–1.89) times higher risk than females. It was highest among children aged 0–4 years (IR 1577.9 per 100,000; 95% CI 1414.9–1740.9). It was higher among urban (IR 826.1 per 100,000; 95% CI 748.1–904.2) than rural residents, with an IRR of 1.07 (95% CI 0.94–1.22). It was also highest among auto-rickshaw, bus, and truck drivers (1632.7 per 100,000; 95% CI 1205.0–2060.4).

**Table 2:** Epidemiological distribution of maxillofacial injuries.

Characteristics	Incidence rate* IR (95% CI)	Injury cases^†^ n (%)	Incidence rate ratio IRR (95%CI)
Total	792.6 (760.3–824.8)	1158 (100%)	–
**Sex**			
Female	596.9 (557.3–636.4)	437 (37.7)	Reference
Male	989.1 (938.1–1040.2)	721 (62.3)	1.66 (1.45–1.89)
**Age (years)**			
0-4	1577.9 (1414.9–1740.9)	180 (15.5)	Reference
5-9	1222.9 (1093.7–1352.1)	172 (14.9)	0.96 (0.63–0.96)
10-17	728.1 (652.2–803.9)	177 (15.3)	0.46 (0.37–0.57)
18-24	546.1 (470.4–620.6)	100 (8.61)	0.35 (0.27–0.45)
25-39	730.8 (671.2–790.5)	288 (24.9)	0.46 (0.37–0.57)
40-59	623.6 (557.9–688.8)	176 (15.2)	0.39 (0.31–0.49)
60+	626.1 (518.4–733.7)	65 (5.59)	0.40 (0.30–0.54)
**Residence**			
Rural	773.9 (717.2–830.8)	728 (62.9)	Reference
Urban	826.1 (748.1–904.2)	430 (37.1)	1.07 (0.94–1.22)
**Occupation**			
Housewife	473.1 (426.9–519.2)	202 (17.4)	Reference
Van/rickshaw driver	1123.3 (775.2–1471.4)	20 (1.73)	2.37 (1.49–3.78)
Auto-rickshaw/ bus/ truck driver	1632.7 (1205.0–2060.3)	28 (2.42)	3.45 (2.29–5.20)
Farmer	678.3 (565.9–790.7)	70 (6.04)	1.43 (1.13–1.81)
Businessman	662.4 (561.0–761.8)	82 (7.08)	1.40 (1.12–1.74)
Construction worker	1027.9 (892.2–1163.8)	110 (9.50)	2.17 (1.76–2.66)
Service holder	678.3 (553.8–802.8)	57 (4.94)	1.43 (1.08–1.88)
Student	862.7 (796.7–728.7)	328 (28.3)	1.82 (1.51–2.20)
Pre-school child	1628.6 (1466.7–1791.8)	194 (16.8)	3.44 (2.78–4.25)
Other	819.3 (680.6–957.9)	67 (5.79)	1.73 (1.31–2.28)
**Place of injury occurrence**			
Playground	41.1 (31.9–52.9)	60 (5.16)	Reference
Within the house	234.1 (210.5–260.2)	342 (29.5)	5.70 (4.33–7.50)
Yard	136.9 (119.2–157.2)	200 (17.3)	3.33 (2.50–4.44)
Roads	262.8 (237.8–290.5)	384 (33.2)	6.40 (4.88–8.40)
Agricultural field	35.6 (27.1–46.7)	52 (4.49)	0.87 (0.6–1.26)
Industry	15.7 (10.5–23.7)	23 (1.99)	0.38 (0.23–0.61)
Construction area	14.4 (9.4–22.0)	21 (1.81)	0.35 (0.21–0.58)
Other^‡^	52.0 (41.6–65.1)	76 (6.55)	1.27 (0.91–1.78)
**Injury aetiology**			
Industrial accident	13.7 (8.83–21.2)	20 (1.87)	Reference
RTAs^§^	203.9 (182.1–228.5)	298 (27.6)	14.9 (9.47–23.4)
Assault^‖^	80.1 (66.8–95.9)	117 (10.9)	5.85 (3.64–9.4)
Falls	216.9 (194.3–242.2)	317 (29.4)	15.8 (10.1–24.9)
Burn	49.9 (39.7–62.8)	73 (6.78)	3.65 (2.23–5.99)
Animal contact	54.8 (43.9–68.2)	80 (7.43)	3.99 (2.45–6.52)
Exposure to a blunt mechanical object^¶^	98.6 (83.7–116.1)	144 (13.4)	7.2 (4.11–11.5)
Suffocation	12.3 (17.8–19.6)	18 (1.68)	0.9 (0.48–1.70)
Other unintentional injuries^#^	6.84 (3.68–12.7)	10 (0.94)	0.5 (0.23–1.07)
**Condition of the injured**			
Conscious	653.6 (613.5–696.4)	955 (82.5)	Reference
Unconscious	138.9 (121.0–159.4)	203 (17.5)	0.21 (0.18–0.24)
**Condition of conscious individuals**			
Ambulant without assistance	492.8 (458.1–530.1)	720 (75.7)	Reference
Ambulant with assistance	84.9 (71.2–101.2)	124 (13.0)	0.17 (0.14–0.21)
Non-ambulant	73.2 (60.6–88.5)	107 (11.3)	0.15 (0.12–0.18)
**Severity of the injury**			
Mild	671.4 (630.7–714.8)	981 (84.7)	Reference
Moderate	84.9 (71.2–101.2)	124 (10.7)	0.13 (0.11–0.16)
Severe	23.9 (17.2–33.4)	35 (3.04)	0.04 (0.03–0.06)
Very severe	12.3 (7.76–19.6)	18 (1.56)	0.02 (0.01–0.03)
**Body regions encountering concomitant injuries**			
Abdomen	10.3 (6.19–17.0)	15 (2.25)	Reference
Head	96.5 (81.8–113.8)	141 (21.4)	9.41 (5.53–16.0)
Eye	19.8 (13.8–28.6)	29 (4.40)	1.93 to (1.03–3.6)
Neck	27.4 (20.1–37.3)	40 (6.05)	2.67 (1.48–4.83)
Chest	71.2 (58.7–86.3)	104 (15.8)	6.94 (4.04–11.9)
Waist	23.3 (16.6–32.6)	34 (5.15)	2.27 (1.24–4.17)
Upper extremity	179.3 (158.7–203.1)	262 (39.8)	17.5 (10.4–29.6)
Lower extremity	23.3 (16.6–32.6)	34 (5.15)	2.27 (1.24–4.17)

Incidence rates (IRs) and incidence rate ratios (IRRs) of maxillofacial injuries stratified by sociodemographic and clinical characteristics among cases (n = 1158) identified in the Bangladesh Health and Injury Survey, 2016.

* Annualised IR = (new cases/ 100,000 person-years)

† Reported over a 6-month recall period.

‡ Other places of injury occurrence included factory, office, trees, school premises, sports facilities, markets/shopping areas, public transport areas, recreational areas, forests, water bodies, workshops/garages, or stairs/rooftops.

§ RTAs included pedestrian or bicycle, two-wheeler, three-wheeler, and four-wheeler vehicle injuries.

‖ Assault included injuries from intentional harm by another person, either direct or indirect.

¶ Exposure to a blunt mechanical object included injuries from contact with non-sharp tools such as machinery parts, building materials, or household items.

# Other unintentional injuries include electrocution, drowning, or chemical exposures.

Roads were the most common locations of injury occurrence (IR 262.8 per 100,000 persons per year; 95% CI 237.8–290.5), with a 6.40 (95% CI 4.88–8.40) times higher risk than the playgrounds. Fall injuries accounted for the highest IR (216.9 per 100,000; 95% CI 194.3–242.2). Injuries yielding unconsciousness had an IR of 138.9 (95% CI 121.0–159.4) per 100,000. Severe (IR 23.9 per 100,000; 95% CI 17.2–33.4) and very severe (IR 12.3 per 100,000; 95% CI 7.76–19.6) cases were fewer. The upper extremities (IR 179.3 per 100,000; 95% CI 158.7–203.1) were most commonly injured along with the face, with a 17.5 (95% CI 10.4–29.6) times higher risk of injury compared to abdominal injuries.

Table 3 presents the healthcare-related characteristics of patients with maxillofacial injuries. Nearly half of the injured did not receive any treatment, whether hospital-based, informal, or first-aid care (IR 373.7 per 100,000; 95% CI 343.6–406.4). Of those who received treatment (52.6%), hospital-admitted cases accounted for an IR of 119.1 (95% CI 94.7–127.5) per 100,000, while the remainder received outpatient or informal care. Patients were mostly admitted to the district hospitals (DHs) (IR 52.0 per 100,000; 95% CI 41.6–65.1). Surgery was the treatment modality for 43.6% of the admitted. Local anaesthesia (LA) was administered in 79.2% of surgeries. The mean OOP expenditure for inpatients was USD 470.5 (SD 1497.1), while the medians of LOS in hospital and work loss time due to maxillofacial injuries were 3 (IQR 2–8) and 5 (IQR 2–10) days, respectively.

**Table 3:** Healthcare-related characteristics and costs associated with maxillofacial injuries.

Variables	Incidence rate^*^ IR (95% CI)	Frequency^†^ (%)/ Mean (SD)/ Median (IQR)	Incidence rate ratio IRR (95%CI)
**Treatment**			
Received	414.8 (383.0–449.1)	606 (52.6)	Reference
Didn’t receive	373.7 (343.6–406.4)	546 (47.4)	0.90 (0.80–1.01)
**Admission to health facilities**			
No	668.7 (628.0–711.9)	977 (84.9)	Reference
Yes	119.1 (94.7–127.5)	174 (15.1)	0.18 (0.15–0.21)
**Type of health facility of admission**			
Upazila health complex (UHC)	33.5 (25.4–44.4)	49 (28.9)	Reference
District hospital (DH)	52.0 (41.6–65.1)	76 (45.0)	1.56 (1.09–2.23)
Private clinic	10.9 (6.71–17.9)	16 (9.50)	0.33 (0.19–0.58)
Specialized hospital	19.2 (13.2–27.8)	28 (16.6)	0.57 (0.36–0.91)
**Surgery among the admitted**			
Not done	66.4 (54.4–81.0)	97 (56.4)	Reference
Done	51.3 (40.9–64.4)	75 (43.6)	0.77 (0.57–1.04)
**Type of anaesthesia used during surgery**			
Local anaesthesia (LA)	39.0 (30.1–50.6)	57 (79.2)	Reference
General anaesthesia (GA)	10.3 (6.24–17.0)	15 (20.8)	0.26 (0.15–0.46)
Mean inpatient OOP expenditure (USD)		470.5 (1497.1)	
Median length of stay (LOS) in hospital (days)		3 (2–8)	
Median time of work loss (days)		5 (2–10)	

Incidence rates (IRs) and incidence rate ratios (IRRs) across healthcare-related characteristics of patients with maxillofacial injuries, from the Bangladesh Health and Injury Survey, 2016. Continuous variables are summarized as mean (SD) or median (IQR), as appropriate.

* Annualised Incidence rate (IR) = (new cases/ 100,000 person-years).

† Reported over a 6-month recall period.

Table 4 presents the estimated and projected national burden of non-fatal maxillofacial injuries in Bangladesh, using proportional extrapolation. Based on BHIS 2016 data and the total population of Bangladesh, approximately 1.27 million injury cases were estimated that year [45]. When extrapolated to the estimated 2026 national population, the projected total burden was about 1.41 million cases, indicating an overall increase across both sexes [45].

**Table 4:** Estimated and projected national burden of maxillofacial injuries, Bangladesh (2016–2026)^*^.

Categories	Estimated new caseload in 2016 (million)	Projected new caseload in 2026 (million)
Total	1.27	1.41
Male	0.78	0.87
Female	0.49	0.54

* New caseloads were estimated using the following formula: [(Incidence rate × total population)/100,000]: [(792.6 × 160,811,932)/ 100,000] ≈ 1,274,595 in 2016, Bangladesh [45]. For 2026, the projected new caseload was: [(Incidence rate × total population)/ 100,000]: [(792.6 × 177,818,044)/ 100,000] ≈ 1,409,317. Similarly, new caseloads for males and females were estimated for both 2016 and 2026.

## Discussion

Although maxillofacial injuries contribute substantially to morbidity and healthcare burden worldwide [2,21], there exists no population-based research on this issue in Bangladesh. To address this gap, this study secondarily analysed the BHIS 2016 dataset and reported annual IRs and IRRs for non-fatal maxillofacial injuries to explore disparities across sociodemographic groups. The sociodemographic distribution in this survey was analogous to Bangladesh’s total population [46,47]. On an important note, the annualised IRs provide actionable evidence for health system capacity planning, as acute injury-related emergencies and healthcare demands are mainly driven by new or incident cases. By underscoring key high-risk groups and treatment gaps, this study’s results can guide targeted injury-prevention strategies, policy reforms to subsidise OOP costs, and resource allocation to improve infrastructure, emergency care, and rehabilitation services in Bangladesh and other LMICs.

The IR of maxillofacial injuries in 2016 was 792.6 per 100,000 population in Bangladesh. In South Asia, the age-standardised incidence rate (ASIR) of facial fractures marginally decreased (1.7%) over a period of 30 years, from 153.1 (95% CI 123.6 to 191.0) per 100,000 in 1990 to 150.5 (95% CI 119.3 to 191.0) per 100,000 in 2019 [2]. Importantly, the decline (14.1%) in ASIR was steeper in global estimates [2]. Notably, ASIRs may drop while absolute new case counts rise due to population growth and demographic changes, and this pattern is plausible given the 2019 global and regional facial fracture data [2]. However, context-relevant epidemiological and healthcare-related data on the full spectrum of maxillofacial injuries in LMICs and the South Asian region remain scarce. We also lack any contemporary national data to assess trends within the country, even from the 2003 BHIS study [38,39]. In light of the global (19.4%) and regional (61.5%) upward trend of facial fractures’ incidence observed from 1990 to 2019 [2], extrapolating the estimated national incidence of non-fatal maxillofacial injuries from the BHIS 2016 to the population in 2026 projected an increase in events by 11.02%—escalating from 1.27 to 1.41 million new cases annually. On that account, the burden of maxillofacial injuries becomes an indisputable public health challenge for Bangladesh. Results showed that males had a 1.65 times higher risk than females. It is concordant with the findings from other countries in South Asia, sub-Saharan Africa, the United States, Europe, and China, albeit the ratios vary [2,20,21,26,48–50]. Males also experience a greater number of fractures in other body parts [51], underscoring their greater occupational hazards, risk-taking behaviours, road usage patterns, and sports [2,21,52]. Nevertheless, some earlier studies showed that older females are more susceptible to facial fractures, potentially attributed to postmenopausal osteoporosis [2,53].

According to this study’s estimates, the IR was higher across the subpopulation of children aged 0–9 years. Many previous studies reported relatively lower rates among children [54–57]. Therefore, a higher rate in our context warrants heightened attention and further research. Critically, craniofacial injuries prevail as a leading cause of morbidity and mortality among children owing to their greater cranial mass-to-body ratio [58]. Prior research evinced that falls are the leading cause in early childhood [54,59], linked to the built environment, earnings, and numerous other sociodemographic factors [60,61]. In South Asia, falls and RTAs accounted for 47% and 32% of reported paediatric injuries, respectively [59]. Community-based parental educational programmes targeting low-income and marginalized families and promoting safeguarded environments for children may lessen the burden [62,63]. Individuals aged 25–39 also had a higher rate, which is consistent with previous studies [48,50,64]. They constitute the primary workforce, escalating their likelihood of exposure to frequent outdoor tasks, road usage, and industrial hazards that elevate risk [64,65].

Urban residents exhibited a higher rate, corroborating prior research [66]. Potential causes include suboptimal compliance with traffic regulations and protective gear use, unsafe infrastructure, and high-speed driving in urban LMIC settings [67]. In harmony with these findings, a high IR among auto-rickshaw, bus, and truck drivers was observed. Previous research suggests that vehicle drivers and occupants can be injured from impacts with steering wheels and windshields during RTAs [68]. On that note, the compulsory use of seatbelts and helmets, crashworthy systems like padded steering wheels and laminated windshields, regular vehicle fitness checks and driving licence scrutiny, effective traffic law enforcement, and road‐safety education programmes can be useful. Our construction workers also experienced a high rate, consistent with worldwide trends [52,69,70]. It was reported that 81% and 87.5% of Bangladeshi workers received no safety training and personal protection equipment (PPE), respectively [71]. Therefore, on-site safety training should be provided alongside the mandatory use of PPE, supported by adequate provision, routine inspections, and prompt access to emergency care.

Following road-related injuries, within-home injuries accounted for the second-highest location-specific incidence rate. Pre-school children, students, and housewives together contributed to a significant percentage of cases. This pattern may reflect a combination of common unintentional domestic trauma, home-safety risks and dense indoor-outdoor exposure, and a contribution from intimate partner violence (IPV). In Bangladesh, this population spends a larger portion of its daily time in the domestic environment, where cumulative exposure to home-based hazards due to structural vulnerabilities, including falls, slips/trips, furniture- or stair-related, and sharp-object injuries, can be higher. Pre-school children may sustain facial injuries by falling from traditional platform beds, or *“Chowkis",* that lack safety guardrails. School-aged children may be affected during indoor-outdoor sports and occasional physical altercations, partly attributable to heightened risk-taking behaviour [72]. On a similar note, the 2024 Violence Against Women Survey found that 46.7% of Bangladeshi women experienced physical violence type IPV in their lifetime, while 64% of victims disclosed it to no one [73]. Plausibly, these violent acts may sometimes result in maxillofacial trauma, which may remain unreported initially but can later be detected in household surveys such as the BHIS 2016. We suggest that community-based educational campaigns could be implemented, ranging from training mothers and caregivers in home hazard-reduction initiatives for young children as part of existing maternal and child health programmes, to disseminating essential information on help-seeking for IPV. For school-aged children, attention to lessening sports-related injuries and risk-taking behaviour is warranted.

Facial trauma research suggests a changing trend in epidemiology over several decades, stemming from urbanisation patterns, socioeconomic structures, customs, crimes, time, and environments across the countries [21,74,75]. This study found that the leading cause of maxillofacial injuries was falls, followed by RTAs, which is a largely observed pattern worldwide [2,48,76]. The risk of falls is higher among older adults, yet it can occur at any age [77,78]. Young adults often encounter it during recreational activities, while older adults may suffer owing to age-related strength decline, coordination impairments, and musculoskeletal disorders [79–82]. However, a prior Bangladeshi study reported that most inpatients’ maxillofacial fractures were attributed to RTAs [27]. Studies from Asia, Africa, and many LMICs reported RTAs as a leading cause, followed mostly by falls, and sometimes assaults [21,22,26,48,50,83]. Key contributors to higher rates of RTAs encompass unsafe infrastructure, increased vehicle ownership and traffic volume, non-compliance with inadequate regulations, and driving obsolete vehicles [36,83–85]. Moreover, many public awareness and prevention initiatives are either insufficient or unsuccessful due to being largely disregarded by the masses [86].

In most cases, affected individuals were conscious and ambulatory following the injuries since those were predominantly of mild severity. Severe forms of injuries were few, but they might lead to disabilities in many instances [76]. Disabilities can also result from delayed access to appropriate surgical treatment [76]. They can impose heavy economic burdens on patients and the health system. In line with previous research, we also found that concomitant injuries mostly affected patients’ upper extremities, heads, and chests [11,14,59,87–90]. Experts conclude that associated injuries in the head and chest should be scrutinized following high-impact mechanisms such as RTAs and falls [7,91,92].

The key to managing a non-fatal maxillofacial injury is ensuring optimal functional and aesthetic recovery in a minimal period [6]. Hence, primary wound repair is crucial [17]. In LMICs, 77.8% of patients undergo treatment delays mostly due to shortages of medical equipment or surgeons [22]. In this study, nearly half of the patients received no treatment, yet an even greater proportion sustained concomitant injuries. Untreated cases may heal incorrectly with visible scars, neurovascular damage, asymmetry, and disfigurement [17,93]. Shockingly, isolated and polytrauma-associated injuries may lead to long-term aesthetic and functional complications even after treatment [94]. These can further induce psychopathological impacts like anxiety, depression, and stigma, and profoundly affect the victim’s social life [94,95]. Nevertheless, this survey was not designed to capture the specific reasons for non-treatment, which may plausibly reflect financial barriers, geographic inaccessibility, perceived mild injury severity, limited health literacy, or other factors. All of these may operate differently across sociodemographic strata in Bangladesh and cannot be determined from these data alone. Further research is imperative to identify the root causes and pertinent actionable strategies.

The public sector dominates healthcare service delivery in Bangladesh [96]. According to the data from BHIS 2016, only 15.1% of patients suffering from non-fatal maxillofacial injuries were treated on an inpatient basis, mostly in secondary-level public facilities such as DHs and UHCs. A Malaysian study reported that 22.9% of patients presenting with isolated injuries and 63.6% with concomitant injuries were treated as inpatients [97]. Two studies from the USA reported that 67.9% of fracture patients, and 87% with only lacerations, were treated as outpatients [98,99]. In contrast to the exemplary hospital settings of these two leading economies, a substantial treatment gap, coupled with limited access to inpatient care and tertiary hospitals, underscores significant health system constraints in our injury-prone context. Additionally, a significant proportion of our inpatients underwent surgery. Only 20.8% of those surgeries were performed under GA, highlighting the degree of injury severity and possible limited availability of operating‑room time and trained anaesthetic personnel in many Bangladeshi hospitals [100]. Future research is needed to examine the shortcomings in treatment trajectories for different facial trauma subtypes across Bangladesh and other LMICs.

Herein, the median LOS in hospital is consistent with studies from India, the UAE, and the USA, while a study from China reported a longer duration [14,33,101,102]. The variation in LOS mostly depends on individuals’ age and severity of injuries [7,103,104]. Days of work loss reflected the substantial productivity loss among injured patients. Additionally, the data revealed that OOP expenditure was over $89 million on inpatient care in 2016. The mean OOP expenditure was lower than the data from Malaysia, Australia, and the USA, but higher than that of sub-Saharan Africa [21,95,105,106]. However, the injury patterns, mechanisms, and timelines varied across these settings. Because of poor infrastructure, unavailability of supplies, and heavy patient load [96], Bangladesh must rely predominantly on low-cost diagnostics and treatments rather than the advanced modalities and high‑end biomaterials used in higher‑income countries. This country’s largely unregulated healthcare system is underfinanced, has low service coverage, and heavily depends on OOP payments [107]. The OOP costs cover 67% of the total health expenditure [108], which may further impoverish our patients. According to the Household Income and Expenditure Survey (HIES) 2016−17, 25% of our population experienced catastrophic health expenditures (CHE), mostly because of OOP costs [109]. Unquestionably, healthcare inequality consistently deprives most underprivileged populations of appropriate healthcare services in many other LMICs [110]. We call for a reformed and well-subsidised health financing system to strengthen treatment facilities and to prioritise disadvantaged populations through education and empowerment, securing access to proper maxillofacial trauma care for all.

Since 2016, Bangladesh has undertaken several injury-related policy initiatives, including the enactment of the Road Transport Act 2018 [111] and the World Bank-financed Bangladesh Road Safety Project in 2024, the first standalone multi-sectoral road safety investment in South Asia [112]. Nevertheless, implementation gaps persist owing to inconsistent and ineffective enforcement and limited institutional surveillance capacity, along with the continued rise in road crash fatalities [111,112]. In this context, the projected 2026 incidence rates from this study remain a pragmatic estimate in the absence of empirically verified nationally representative data.

## Strengths and limitations

This is the first large-scale assessment of the full spectrum of non-fatal maxillofacial injuries in Bangladesh, providing actionable epidemiological and healthcare-related evidence to inform prevention policies and resource planning in LMIC contexts. The BHIS 2016 was conducted on a nationally representative sample using rigorous multistage cluster sampling with PPS methodology and distinct urban and rural sampling units. This methodology produced a self-weighted sample in which each subunit had an equal probability of being selected, eliminating the need for reweighting [113]. Its large sample size accurately depicted the epidemiological landscape and healthcare burden of non-fatal maxillofacial injuries across our sociodemographic groups, yielding generalisable estimates. The household-based approach allowed for estimating both treated and untreated cases, OOP costs, and productivity losses. Importantly, the study findings can guide comparable settings in understanding the complex patterns and challenges associated with this injury category.

This study has several limitations. Reliance on a six‑month recall period might introduce recall bias, resulting in both under- and over-reporting of injuries within this timeframe. However, to mitigate this, data collectors requested the other family members to confirm the injury-related data during documentation. Congruently, the study captured non-fatal maxillofacial injury events only. The BHIS 2016 mortality module recorded fatal injury events by mechanism rather than by anatomical region, making it impossible to isolate deaths occurring among persons who sustained maxillofacial injuries as a distinct epidemiological category. It is also important to note that, as maxillofacial injuries can co-occur with other body-part injuries in fatal polytrauma cases, cause of death attribution to a specific anatomical region is not feasible without postmortem confirmation. Consequently, mortality due to maxillofacial trauma could not be assessed. Evidently, the total injury burden, including fatalities, would likely exceed the estimates presented here. Furthermore, the clinical accuracy of primary respondent- or caregiver-reported data on injury incidence, characteristics, and treatment details could not be ensured, which might introduce bias in reporting and classification. It is also established that reliance on data reported by proxy respondents, such as the household-heads or other respondents instead of the injured person, is a potential limitation of community-based injury surveys [114]. However, as stated earlier, data collectors asked other family members to verify the reported cases and associated information, which likely helped minimize this limitation. On a related note, the BHIS 2016 dataset did not capture clinical subcategories such as isolated soft tissue injuries and/or fractures, preventing stratified estimation of incidence rates, LOS in hospital, and inpatient OOP expenditures. Besides, due to the unavailability of updated national survey data, the projected estimates (in Table 4) assume that the age- and sex-specific incidence rates observed in 2016 remain constant through 2026. Therefore, these figures should be interpreted as rough estimates obtained by proportional extrapolation, intended to establish a planning baseline rather than definitive forecasting. Population-level injury prevention interventions implemented after 2016 could reduce the incidence below the projected values, whereas their absence could increase it. Moreover, while the survey captured both first and subsequent injury events (if any), the BHIS 2016 dataset lacked the stratification during data entry required to later distinguish between single and multiple injuries within the same individual. Consequently, we did not have the categorical data to present single and multiple injuries separately. Instead, we presented an overall incidence rate to quantify the event-based injury burden, irrespective of whether the injuries were single or multiple in nature. Finally, as the survey was cross-sectional, long‑term functional, aesthetic, or financial trajectories beyond the initial injury events could not be assessed. We underscore the need for future research to document the clinical subclassification of non-fatal maxillofacial injuries, assess their healthcare and financial burdens, examine specific occupational hazards, and evaluate long-term outcomes.

## Recommendations

To address the high burden of maxillofacial injuries in Bangladesh, we call for intensified, multifaceted injury prevention efforts, including stricter enforcement of road safety laws and targeted fall prevention programmes. Trauma-care capacity should be expanded by increasing the number of well-equipped secondary healthcare facilities with standardised operating rooms and anaesthesia services, and by strengthening coordination through regular cross-disciplinary training for doctors. Health-financing policy reforms should provide financial security by introducing targeted subsidies, insurance schemes, and cost-effective treatment protocols to reduce OOP expenses. In resource-constrained LMIC settings like ours, integrating these actions into national health strategies can effectively decrease inequities across all levels of care and guide clinicians and policymakers toward more effective trauma management and resource allocation.

## Conclusion

Battling against injuries is now a worldwide challenge. Comprehensive epidemiological data on the injury spectrum are imperative for guiding targeted strategies and implementation. This study depicted the substantial burden and socioeconomic impacts of non-fatal maxillofacial injuries in Bangladesh and highlighted critical gaps in the healthcare infrastructure. This study also deduces that Bangladesh requires robust action plans to strengthen its public health systems to reduce the injury burden. Given that similar gaps exist across many LMICs, these findings convey broader implications for regional and global contexts. Evidently, ensuring safe infrastructure, sustainable injury prevention and rehabilitation policies, integration of injury surveillance into national health information systems, access to emergency care, and attention to socioeconomic consequences are pivotal steps for LMICs to mitigate the growing burden of maxillofacial trauma.

## Supporting information

S1 File Full questionnaire (the original Bengali questionnaire with English translations). The questionnaire used in the Bangladesh Health and Injury Survey (BHIS), 2016.
(PDF)

S2 File Determination of the sample size. (DOCX)
