# Supplementary material for: Epidemiology and healthcare burden of non-fatal maxillofacial injuries in Bangladesh: Evidence from Bangladesh Health and Injury Survey (BHIS), 2016
Source: PLoS One. 2026 Jul 9;21(7):e0353183. doi: 10.1371/journal.pone.0353183 (PMC13349135; doi:10.1371/journal.pone.0353183)

Bangladesh Health and Injury Survey

Household identification number

	City corporation	District headquarters	District rural upazila
**Area**	**1. City Corporation** ☐	**2. District Town** ☐	**3. Rural** ☐
**District/District HQ**	01. Dhaka, 02. Chittagong<br>03. Rajshahi, 04. Khulna,<br>05. Barisal 06. Sylhet,<br>07. Rangpur, 08. Mymensing<br>☐☐	02. Rajbari, 03. Sherpur, 04. Bagerhat,<br>05 Jessore, 07. Joypurhat, 08. Pabna,<br>11. Bhola, 12.Chandpur, 13.Khagrachari<br>14. Kurigram 15. Thakurgaon 16. Sirajgonj,<br>17. Manikgonj, 18. Jhenaidah, 19. Habignj,<br>20. Barguna.<br>☐☐	01. Dhaka, 02. Rajbari, 03. Sherpur,<br>04. Bagerhat, 05. Jessore, 06. Khulna,<br>07. Joypurhat, 08. Pabna, 09. Sylhet,<br>10. Barisal, 11 Bhola, 12. Chandur,<br>13. Khagrachari 14.Kurigram,<br>15. Thakurgaon, 16. Sirajgonj<br>☐☐
**Ward/ Upazilla**	**WARD**<br>☐☐	**WARD**<br>☐☐	**Upazilla name**<br>01. Dhamrai 02. Baliakandi,<br>03.Nalitabari, 04. Fakirhat,<br>05 Chowgacha, 06. Koira,<br>07. Kalai, 08. Santhia,<br>09. Belkunchi, 10. Golapgonj,<br>11. Banaripara 12. Burhanuddin<br>13. Kanchua, 14. Khagrachari Sadar<br>15. Kurigram Sadar, 16. Ranishankail<br>☐☐
**Village/Moholla**	☐☐	☐☐	☐☐
**HOUSEHOLD NO**	☐☐	☐☐	☐☐
**HH** unique code	☐ ☐☐☐☐☐☐☐☐	☐ ☐☐☐☐☐☐☐☐	☐ ☐☐☐☐☐☐☐☐

Module I

Face Sheet (Household and Socio-Economic Information)

	নাম Name	কোড Code	
TYPE OF SURVEY LOCATION এরিয়া	1. City Coorporation<br>2. District Town<br>3. Rural (Upazila)	☐	
CITY CORPORATION	01. Dhaka, 02. Chittagon<br>03. Rajshahi 04. Khulna<br>05. Barisal 06. Sylhet<br>07. Rangpur 08. Mymensing	☐☐	
WARD		☐☐	
MOHALLA		☐☐	
খানার নম্বর HOUSEHOLD NO		☐☐	
জেলা DISTRICT TOWN	02. Rajbari, 03. Sherpur, 04. Bagerhat,<br>05 Jessore, 07. Joypurhat, 08. Pabna,<br>11. Bhola, 12. Chandpur, 13. Khagrachari<br>14. Kurigram, 15. Thakurgaon, 16. Sirajgonj,<br>17. Manikgonj, 18. Jhenaidah, 19. Habignj,<br>20. Barguna.	☐☐	
WARD		☐☐	
MOHALLA		☐☐	সাক্ষাৎকার
খানার নম্বর HOUSEHOLD NO		☐☐	শুরু হবার সময়:
District (village)	01. Dhaka, 02. Rajbari, 03. Sherpur,<br>04. Bagerhat, 05. Jessore, 06. Khulna,<br>07. Joypurhat, 08. Pabna, 09. Sylhet,<br>10. Barisal, 11 Bhola, 12. Chandur,<br>13. Khagrachari, 14.Kurigram,<br>15. Thakurgaon, 16. Sirajgonj	☐☐	|__|__|:|__|__|
উপজেলা UPAZILA	01. Dhamrai, 02. Baliakandi, 03.Nalitabari,<br>04. Fakirhat, 05 Chowgacha, 06. Koira,<br>07. Kalai, 08. Santhia, 09. Belkunchi,<br>10. Golapgonj, 11. Banaripara,<br>12. Burhanuddin, 13. Kanchua,<br>14. Khagrachari 15. Kurigram Sadar,<br>16. Ranishankail	☐☐	সাক্ষাৎকার<br>শেষ করার সময়
গ্রাম VILLAGE/ WARD		☐☐	|__|__|:|__|__|
খানার নম্বর HOUSEHOLD NO		☐☐	
HOUSEHOLD UNIQUE CODE	`	☐ ☐☐ ☐☐ ☐☐ ☐☐	
IInterviewer name and			
Data collector unique code		☐☐	
গত 24মাসে খানায় মৃতের সংখ্যা (কেউ মারা না গিয়ে থাকলে 00 লিখুন):		☐☐	

Section A: Usual (live) household member/s and detection of injury morbidity in last 6 months

This section contains basic information on all live household members

Ind. no.	নাম	খানা প্রধানের সাথে সম্পর্ক	জন্ম তারিখ (দিন/মাস/বছর)	১ বা ততোধিক বছরের ক্ষেত্রে বছরে লিখুন	বয়স ১ বছরের নিচে, মাসে লিখুন	লিঙ্গ পুরুষ -1 মহিলা -2 হিজড়া- 3	বৈবাহিক অবস্থা	লেখাপড়া (সর্বোচ্চ শ্রেণী পাশ)	প্রধান পেশা	ধুমপান করেন কিনা হ্যাঁ - 1 না - 2	পান খান কিনা ? হ্যাঁ - 1 না - 2	সাঁতার জানেন কিনা? হ্যাঁ - 1 না - 2	(নাম) এর কি গত ৬ মাসে কখনো ইনজুরি হয়েছিল? (ইনজুরি নোটিফিকেশান ফর্মে -১ এ তথ্য লিপিবদ্ধ করুন) হ্যাঁ -1, না -2	যদি হ্যাঁ হয়, তাহলে কতবার? যদি হ্যাঁ হয়, মরবিডিটি ফর্ম পুরণ করুন
A01	A02	A03	A04	A05	A 6	A07	A08	A09	A10	A11	A12	A13	A14	A15
1														
2														
3														
4														
5														
6														
7														
8														
9														
10														
11														
12														

- A03- খানা প্রধানের সাথে সম্পর্ক : 01= নিজ (খানা প্রধান) , 02=বাবা, 03= মা , 04= ভাই/বোন , 05= চাচা/মামা/খালু/ফুপা , 06= চাচী/মামী/খালা/ফুপু , 07=শ্বশুর/শ্বাশুরী 08=ছেলে 09=মেয়ে , 10=স্বামী/স্ত্রী , 11= জামাতা/পুত্রবধূ , 12=দাদা/দাদী/নানা/নানী ,13= নাতি/নাতনী , 14= শ্যালক/দেবর/ভাসুর/ভায়রা/ভগ্নীপতি/ শ্যালিকা/ননদ , 15= গৃহকর্মী, 16= ভাতৃ/ভগ্নী-পুত্র/কন্যা , 97= অন্যান্য
- A08-বৈবাহিক অবস্থা : 1= বিবাহিত , 2= অবিবাহিত 3=তালাকপ্রাপ্তা), 4=বিধবা, 5=বিচ্ছিন্ন/ আলাদা ।
- A09- লেখাপড়া : সর্বোচ্চ শ্রেণীপাশ -সাধারণ এবং মাদ্রাসা, 33= মক্তব ; 99= জানা নেই
- A10 -পেশা : 01=কৃষিকাজ , 02=ব্যবসা , 03= ছাত্র , 04= গৃহিণী , 05= চাকুরী , 06=বেকার , 07=দক্ষ শ্রমিক , 08=অদক্ষ শ্রমিক , 09= গৃহকমী , 10= অবসরপ্রাপ্ত , 11=ভ্যান/রিকশা চালক , 12= সিএনজি/বাস/ট্রাক চালক , 14= অন্যান্য , 15 = শিক্ষক , 97=৬ বছরের কমবয়সী ; 77= প্রযোজ্য নয় ;.
- A14-ইনজুরি : **নিম্নবর্ণিত যে কোন ইনজুরি যার জন্য চিকিৎসা সেবা নিতে হয়েছিল অথবা পানিতে ডুবা :** ১. আত্মহত্যার প্রচেষ্টা , ২. পরিবহন দুর্ঘটনা , ৩. সহিংসতা , ৪. পড়ে যাওয়া , ৫. ধারালো বস্তুর আঘাত, ৬. পুড়ে যাওয়া , ৭. পানিতে ডুবা , **৮. দুর্ঘটনাজনিত বিষক্রিয়া ,** ৯. মেশিন/যন্ত্রপাতির দ্বারা জখম , ১০-বিদ্যুতস্পৃস্ট , ১১.প্রাণী/কীটপতঙ্গের দ্বারা জখম , ১২.ভোঁতা বস্তু দ্বারা আঘাত , ১৩. শ্বাসরোধ

Section B: Household member who died in the last 24 months (গত 24 মাসে খানায় মৃতের সংখ্যা (কেউ মারা না গিয়ে থাকলে 00 লিখুন):

This section contains basic information on all deceased household members those who died in the past 24 months

Sl. no	নাম	খানা প্রধানের সাথে সম্পর্ক	মৃত্যুর সময় বয়স ১ বা ততোধিক বছরের ক্ষেত্রে বছরে লিখুন	বয়স ১ বছরের নিচে, মাসে লিখুন	জন্ম তারিখ (দিন/মাস/বছর)	লিঙ্গ পুরুষ -1 মহিলা -2 হিজড়া- 3	বৈবাহিক অবস্থা	লেখাপড়া (সর্বোচ্চ শ্রেণী পাশ)	প্রধান পেশা	মৃত্যুর তারিখ(মৃত্যুর নোটিফিকেশন ফর্ম পূরন করুন)
B01	B02	B03	B04	B05	B06	B07	B08	B09	B10	
91|										
92|										
93|										
94|										
95|										
96										
97										
98										
99										

- B03- খানা প্রধানের সাথে সম্পর্ক 01= নিজ (খানা প্রধান) , 02=বাবা , 03= মা , 04= ভাই/বোন , 05= চাচা/মামা/খালু/ফুপা , 06= চাচী/মামী/খালা/ফুপু , 07=শ্বশুর/শ্বাশুরী , 08=ছেলে , 09=মেয়ে , 10=স্বামী/স্ত্রী , 11= জামাতা/পুত্রবধূ , 12=দাদা/দাদী/নানা/নানী, 13= নাতি/নাতনী , 14= শ্যালক/দেবর/ভাসুর/ভায়রা/ভগ্নীপতি/ শ্যালিকা/ননদ , 15= গৃহকর্মী , 16= ভাতৃ/ভগ্নী-পুত্র/কন্যা , 97= অন্যান্য
- B08-বৈবাহিক অবস্থা : 1= বিবাহিত , 2= অবিবাহিত , 3=তালাকপ্রাপ্তা , 4=বিধবা , 5=বিচ্ছিন্ন/ আলাদা , ।
- B09- লেখাপড়া : সর্বোচ্চ শ্রেণীপাশ -সাধারণ এবং মাদ্রাসা , 33= মক্তব ; 99= জানা নেই
- B10 -পেশা : 01=কৃষিকাজ , 02=ব্যবসা , 03= ছাত্র , 04= গৃহিণী , 05= চাকুরী , 06=বেকার , 07=দক্ষ শ্রমিক , 08=অদক্ষ শ্রমিক 09= গৃহকমী , 10= অবসরপ্রাপ্ত , 11=ভ্যান/রিকশা চালক , 12= সিএনজি/বাস/ট্রাক চালক , 14= অন্যান্য, 97=৬ বছরের কমবয়সী ; 77= প্রযোজ্য নয় .

Section C: Household Characteristics

This section contains some information on the household

NO.	QUESTIONS AND FILTERS	CODING CATEGORIES	SKIP
C01	এই খানার অধীনে বসতভিটায় (পুকুর ও ডোবাসহ) কি পরিমান জমি (ডেসিমাল-এ) আছে?	বসতভিটা (পুকুর ও ডোবাসহ) …....|____|____||____|____| জানি না (Don't know)………………………………… 99	
C02	এই খানার অধীনে কি পরিমান চাষযোগ্য জমি (ডেসিমাল-এ) আছে?	চাষযোগ্য জমি (Agricultural land)...|____|____||____|____| জানি না (Don't know)………………………………… 99	
C03	বসত ঘরের ছাদের প্রধান নির্মাণ সামগ্রী<br>*[সাক্ষাতকার গ্রহনকারী:দেখে লিখুন]*	<u>প্রাকৃতিক ছাদঃ Natural Roof</u><br>ছাদ নেই No roof ....01<br>খড়/ছন/তালপাতাThatch/Palm leaf ....02<br><u>কাঁচাছাদঃ Rudimentary Roof</u><br>বাঁশ Bamboo ....03<br>কাঠের তক্তা Wood Planks ....04<br>কার্ডবোর্ড Cardbord ....05<br><u>উন্নতছাদঃ Finished roof (Pukka)</u><br>টিন Tin ....06<br>কাঠ Wood...07<br>সিরামিক টাইলস Ceramic Tiles ....08<br>সিমেন্ট Cement/Concrete ....09<br>টালি Tiles..10<br>কাষ্ঠ খন্ড (টালি/ শ্লেঠ) Roofing Shingles ....11<br>অন্যান্য (নির্দিষ্ট করুন) Others (Specify)..................................... 97	
C04	বসত ঘরের মেঝের প্রধান নির্মাণ-সামগ্রী<br>[সাক্ষাতকার গ্রহনকারী: *দেখে লিখুন]*	<u>কাঁচা মেঝে Natural floor</u><br>মাটি/বালু Earth/sand ....01<br><u>প্রাথমিক পর্যায়ের মেঝেঃ Rudimentary floor</u><br>কাঠের তক্তা Wood Planks ....02<br>তালগাছ/ বাঁশ Palm/Bamboo ....03<br><u>উন্নত মেঝে Finished floor</u><br>নকশা করা কাঠের পাটাতন/পালিশকৃত কাঠ Parquet/Polished Wood...04<br>সিমেন্ট/ইটবালি জমানো Cement/Concrete ....05<br>সিরামিক টাইলস/মোজাইক Ceramic Tiles/Mosaic ....06<br>কার্পেট Carpet ....07<br>অন্যান্য(নির্দিষ্ট করুন) Others (specify)...................................... 97	

NO.	QUESTIONS AND FILTERS	CODING CATEGORIES	SKIP
C05	বসত ঘরের দেয়ালের প্রধান নির্মাণ-সামগ্রী<br>[সাক্ষাতকার গ্রহনকারী: *দেখে লিখুন]*	<u>প্রাকৃতিক দেয়ালঃ Natural Walls</u><br>দেয়াল নাই No walls ....01<br>পাটকাঠি/বেত/তালগাছ/গাছের গুঁড়ি Cane/Palm/Trunks ....02<br>মাটি Mud..03<br><br><u>প্রাথমিক পর্যায়ের দেয়ালঃ Rudimentary Walls</u><br>মাটিসহ বাঁশ Bamboo with Mud..04<br>মাটিসহ পাথর Stone with Mud ....05<br>প্লাই-উড Plywood ....06<br>কার্ডবোর্ড Cardbord ....07<br><br><u>উন্নত দেয়ালঃ Finished Walls</u><br>টিন Tin..08<br>ইট / সিমেন্ট (প্লাস্টারসহ) Bricks/ Cement (With plaster) ....09<br>চুনসুড়কি দিয়ে তৈরী পাথরের দেয়াল (Stone with lime) ....10<br>ইট (প্লাস্টার ছাড়া) Bricks (Without plaster)..........................11<br>উন্নতমানের কাঠের তক্তা Polished Wood Planks/Shingles........12<br>অন্যান্য(নির্দিষ্ট করুন) Others (specify)___________ 97	
C06	আপনাদের খানায় কয়টি শোবার ঘর আছে?	শোবার ঘরের সংখ্যা (Number of bedrooms)… …...|____|____|	
C07	আপনার খানার সদস্যদের খাবার পানির প্রধান উৎস কি?	<u>পাইপের পানিঃ (Piped water)</u><br>বাড়ীর ভিতরে ট্যাপের (পাইপের) পানি (Piped inside dwelling) ....01<br>বাড়ীর বাহিরে/উঠানে ট্যাপের (পাইপের) পানি (Piped into yard/plot)...02<br>ট্যাপের (পাইপের) পানি (Public tap/standpipe)...........................03<br>টিউবওয়েল (Tubewell or borehole)..04<br><u>কুপের পানিঃ (Dugwell)</u><br>সংরক্ষিত কুপ(Protected well)..05<br>অসংরক্ষিত কুপ(unprotected well) ....06<br><u>ঝরণার পানিঃ (Water from spring)</u><br>সংরক্ষিত ঝরণার পানি (Protected spring)...................................07<br>অসংরক্ষিত ঝরণার পানি (unprotected spring)..............................08<br>বৃষ্টির পানি (Rain water) ....09<br>ট্যাঙ্কের পানি (Tanker truck) ....10<br>ছোট ট্যাঙ্কের পানি (cart with small tank) ....11<br>পুকুর/খাল/বদ্ধ জলাশয়/হ্রদ/দীঘি/বিল/হাওড় (Surface water:river/dam/lake/Pond/irrigation channel) ....12<br>বোতলের পানি(bottled water) ....13<br>অন্যান্য (নির্দিষ্ট করুন) Other (Specify)___________ 97	

NO.	QUESTIONS AND FILTERS	CODING CATEGORIES	SKIP
C08	আপনাদের খানার সদস্যরা সাধারনত কোন ধরনের পায়খানা ব্যবহার করেন?<br><br>[সাক্ষাতকার গ্রহনকারী:*দেখে লিখুন*]	ফ্ল্যাশ ল্যাট্রিন/ পায়খানা (Flush or pour flush toilet)<br>ফ্ল্যাশ করে পাইপের মাধ্যমে অপসারন (Flush to piped sewer system)....01<br>ফ্ল্যাশ করে সেপটিক ট্যাঙ্ক এ ধারন (Flush to septic tank).................02<br>ফ্ল্যাশ করে গর্তে ধারন Flush to pit latrine)..................................03<br>ফ্ল্যাশ করে পাইপের মাধ্যমে অপসারন (Flush to somewhere else).....04<br>ফ্ল্যাশ করে অজানা স্থানে অপসারন (Flush, don't know where) ....05<br><br>গর্তের (পিট) ল্যাট্রিন(Pit latrine)<br>পিট ল্যাট্রিন (স্লাবসহ) (Pit latrine with slab) ....06<br>পিট ল্যাট্রিন (স্লাবছাড়া)/ খোলা গর্ত (Pit latrine without slab/open pit)..07<br>কম্পোস্টিং ল্যাট্রিন (Composting latrine)...................................08<br>বালতি টয়লেট (Bucket toilet)...09<br>খোলা/ ঝুলন্ড় ল্যাট্রিন (Hanging latrine/Hanging toilet).............10<br>ল্যাট্রিননাই/ঝোপ ঝাড়/মাঠ (No facility/Bush/Field)....................11<br>অন্যান্য (নির্দিষ্ট করুন) Others (Specify) 97	
C09	আপনাদের খানায় এই জিনিসগুলো আছে কি?<br>[প্রত্যেকটি জিনিসের কথাই জিজ্ঞেস করুন]	হ্যাঁ Yes না No<br>রেডিও (Radio).. 1......2<br>টেলিভিশন (Television).. 1.......2<br>ডিসের লাইন Dishre line .... 1……2<br>মোবাইল ফোন (mobile phone)....................................... 1.......2<br>টেলিফোন (Land Telephone)... 1.......2<br>ফ্রিজ (Refrigerator)... 1.......2<br>বাইসাইকেল (Bicycle).. 1.......2<br>মোটরসাইকেল/স্কুটার (Motor cycle/Scooter)..................... 1.......2<br>ভিসিপি/ভিসিআর/ডিভিডি (VCR/VCP/DVD player)............ 1.......2<br>বৈদ্যুতিক পাখা (Electric Fan)... 1.......2<br>গাড়ি/মাইক্রোবাস (Car/Microbus).................................... 1.......2<br>নৌকা/ইঞ্জিনচালিত নৌকা (boat/Motor-Boat)...................... 1.......2<br>পানির পাম্প (Water pump)... 1.......2<br>আলমিরা (Almira/wardrobe).. 1.......2<br>টেবিল (Table)... 1.......2<br>চেয়ার (Chair)... 1.......2<br>কম্পিউটার Computer.. 1……2<br>বিছানা Bed…………………………………………….1……2<br>সোফা Sofa…………………………………………….1……2	01<br>02<br>03<br>04<br>05<br>06<br>07<br>08<br>09<br>10<br>11<br>12<br>13<br>14<br>15<br>16<br>17<br>18<br>19
C10	আপনার খানায় কি বিদ্যুৎ আছে?	হ্যাঁ Yes ....<br>না No..	1<br>2
C.11	পরিবারের মাসিক আয়	টাকার পরিমান (Amount)…………………………………..	

Module II Section D: Death Confirmation

House hold indentificatio number	☐ ☐☐☐☐☐☐☐☐
Person Number	☐☐
Name of the person:	

এই অংশে খানার মৃত সদস্য সংক্রান্ত তথ্য রয়েছে This section contains information on deceased household member

NO.	QUESTIONS AND FILTERS	CODING CATEGORIES	SKIP
D01	মৃত্যুর তারিখ Date of death: (dd/mm/yyyy)	|___|___| |___|___| |___|___|___|___|	
D02	লিঙ্গ Sex of the deceased person	পুরুষ Male… 1<br>মহিলা Female… 2<br>হিজড়া (Transgender) … 3	
D03	মৃত্যুর স্থান Place of death	নিজ বাড়ি Home… 01<br>হাসপাতাল Hospital… 02<br>কর্মক্ষেত্র Workplace… 03<br>যাত্রাপথে On the way… 04<br>অন্যান্য (নির্দিষ্ট করুন) Others specify)____________ …97	
D04	শেষ অসুস্থতার সময়ের লক্ষণ ও উপসর্গ (আঘাত/ইনজুরি ব্যতীত মৃত্যুর ক্ষেত্রে)<br>(প্রতিটি প্রশ্ন জিজ্ঞাসা করুন)<br>Symptoms and signs of final illness (for non-injury deaths)<br>[Ask each question]	হ্যাঁ (yes) না(No) জানিনা(DK)<br>জ্বর? Did s/he have a fever? … 01……02……99<br>র‍্যাশ/ফুসকুড়ি? Did s/he have a rash? … 01……02……99<br>দৈহিক বৃদ্ধি কি স্বাভাবিক ছিল?<br>Was physical growth normal? … 01……02……99<br>বিবর্নভাব(অ্যানিমিয়া)/ জন্ডিস? Sign of pallor/jaundice? … 01……02……99<br>কাশি? Did s/he have a cough? … 01……02……99<br>শ্বাসজনিত কোনো সমস্যা? Any respiratory problem? … 01……02……99<br>পাতলা পায়খানা/ডায়রিয়া? Did s/he have diarrhoea? … 01……02……99<br>বমি? Did s/he have vomiting? … 01……02……99<br>পেটে ব্যাথা? Any abdominal pain? … 01……02……99<br>পেট ফাঁপা? Any abdominal distension? … 01……02……99<br>ওজনহ্রাস? Any weight loss? … 01……02……99<br>বুকে ব্যাথা? Any chest pain? … 01……02……99<br>পিন্ড? Any mass? … 01……02……99<br>মাথা ব্যাথা? Any headache? … 01……02……99<br>ঘাড় শক্ত? Any stiff neck? … 01……02……99<br>খিঁচুনী? Any convulsion? … 01……02……99<br>মূর্ছা/অজ্ঞান? Any fits/unconsciousness? … 01……02……99<br>পক্ষাঘাত? Any paralysis? … 01……02……99<br>প্রশ্রাবের রং কি স্বাভাবিক ছিল? Normal urine colour? … 01……02……99<br>সার্জারী/অস্ত্রপোচার? Any surgery/operation? … 01……02……99<br>অন্যান্য (নির্দিষ্ট করুন)Others specify)__________ 01 ……02……99	
D05	মৃত ব্যক্তির উলে-খিতঅসুস্থতাগুলোর কোনটি ছিল কিনা জিজ্ঞাসা করুন(প্রতিটি প্রশ্ন জিজ্ঞাসা করুন)	উচ্চ রক্তচাপ Hypertension … 01……02……99<br>হৃদরোগ Heart disease … 01……02……99<br>বহুমুত্র Diabetes … 01……02……99<br>ক্যানসার Cancer … 01……02……99	
D06	মৃত্যুর পূর্বে কতদিন অসুস্থ ছিল?	|____|____|____| দিন days	
D07	কি কারনে মৃত্যু হয়েছিল?	ইনজুরিজনিত মৃত্যু (Injury related death)… 01<br>অসুস্থতা জনিত মৃত্যু (Death due to illness /non-injury cause)….. 02	

<u>সাক্ষাতকার গ্রহনকারী (Interviewer):</u>

- নিশ্চিত হোন মৃত্যু আঘাত/ইনজুরির কারনে হয়েছিল কি না ? 1= হ্যাঁ (Yes) 2= না (No)
- যদি মৃত্যু আঘাত/ইনজুরির কারনে হয়ে থাকে তাহলে ইনজুরি মর্টালিটি ফর্ম (Module VI) পূরন করুন।

Module III , Section D: Information on injury morbidity

Household unique number □ □□□□□□□□

Person number □□

Name of the person:

No.	Questions	Coding Categories		
E 01.	ইনজুরিজনিত অসুস্থ ব্যাক্তির লিঙ্গ	পুরুষ Male…………………………………………………………………<br>মহিলা Female……………………………………………………………..<br>হিজড়া Hizra (Transgender) ……………………………………………	1<br>2<br>3	
E 02.	কিভাবেইনজুরিহয়েছিল ?<br><br>নির্দিষ্ট ইনজুরিপদ্ধতিফর্মপূরণকরুন	আত্মহত্যা-এম ১ Attempt to suicide/suicide-M1……………………………..<br>সড়ক দুর্ঘটনা-এম ২ Transport injury- M2………………………………………<br>সহিংসতা-এম ৩ Violence-M3……………………………………………………<br>পড়েযাওয়া-এম ৪ Fall-M4…………………………………………………….<br>ধারালো বস্তু দ্বারা কেটে যাওয়া-এম ৫ Cut injury-M5…………………………..<br>পুড়েযাওয়া-এম ৬ Burn-M6………………………………………………<br>ডুবেযাওয়া-এম ৭ Drowning-M7…………………………………………..<br>দুর্ঘটনাজনিতবিষপান-এম ৮ Unintentional poisoning –M8………………………<br>মেশিন/যন্ত্রপাতিরআঘাত-এম ৯ Machine injury -M9…………………………….<br>বিদ্যুৎস্পৃষ্ট -এম ১০ Electrocution-M10……………………………………..<br>প্রানী ও কীট পতঙ্গের কামড় / আঘাত-এম ১১ Animal bite injury-M11……………<br>ভোঁতা বস্তুর আঘাত -এম ১২Injury by blunt object-M12…………………………<br>দুর্ঘটনাজনিতশ্বাসরোধ-এম ১৩ Suffocation-M13……………………………<br>অন্যান্য (উল্লেখকরুন) Others (Specify) ………………………………...<br>জানা নেই Don't know…………………………………………………...	01<br>02<br>03<br>04<br>05<br>06<br>07<br>08<br>09<br>10<br>11<br>12<br>13<br>97<br>99	
E 03.	কত তারিখে ইনজুরি ঘটেছিল ?	D D M M Y Y Y Y		
E 04.	কখন ইনজুরি ঘটেছিল ?(২৪ ঘন্টায় লিখুন)	H H M M		
05.	আঘাতপ্রাপ্তিরসময় ব্যক্তিটি কোথায়ছিল?	শোয়ার ঘর Bed room…………………………………………………………<br>বসার ঘর Living room……………………………………………………….<br>রান্না ঘর Kitchen…………………………………………………………<br>গোসল খানা/পায়খানা Bathroom…………………………………………….<br>বাড়ীর উঠান Yard……………………………………………………………<br>বারান্দা Verenda ……………………………………………. ………………..<br>এক কক্ষ বিশিষ্ট ঘর One room dwelling…………………………………………<br>শ্রেণীকক্ষ Class room……………………………………………………..<br>স্কুলের খেলার মাঠ School play ground………………………………………<br>শিক্ষা প্রতিষ্ঠানের হোস্টেল Hostel of educational institute……………………<br>অন্য খেলাধুলার স্থান/ ক্রীড়াঙ্গন Other playground……………………………<br>রাস্তায় /মহাসড়কে Roads/highway…………………………………………<br>রেলষ্টেশন/ফেরী/লঞ্চ ঘাট/বাসস্ট্যান্ড/অন্য পরিবহন এলাকা Railway station/Ferry/ Launch station<br>কৃষিক্ষেত্র/খামার বাড়ী/চাতাল Agricultural field…………………………………<br>শিল্প/কলকারখানা/ওয়ার্কশপ Industry/factory/workshop………………………..<br>জলাশয় Water reservoir..…………………………………………………	01<br>02<br>03<br>04<br>05<br>06<br>07<br>08<br>09<br>10<br>11<br>12<br>13<br>14<br>15	

		হাট/ বাজার/ Market/Bazaar…………………………………………….........<br>অফিস Office………………………………………………………………..<br>নির্মাণ এলাকা Construction area…………………………………………….<br>অন্যান্য (উল্লেখকরুন) Others (Specify) ________________________<br>জানা নেই Don't Know………………………………………………………	16<br>17<br>18<br>19<br>97<br>99	
E 06.	ইনজুরির অভিপ্রায় কিছিল ?	অনিচ্ছাকৃত Unintentional……….. …………………….<br>ইচ্ছাকৃত নিজের ক্ষতি Intentional/Self harm…………………<br>সহিংসতা Assault/Violence…………………………….....<br>নির্ণয় করা যায় নি Undetermined……………………...........	01<br>02<br>03<br>04	

E 07. ব্যক্তিটির শরীরের কোন কোন অঙ্গে এবংকি ধরনের ইনজুরি হয়েছিল?

শরীরের কোন অঙ্গে	ক্ষত অঙ্গ	ইনজুরি ধরনের কোড
1. মাথা Head		
2. মুখমন্ডল Face		
3. চোখ Eye		
4. ঘাড় Neck		
5. বুক/পিঠ Chest		
6. পেট Abdomen		
7. বাহু হাত ব্যতীত Upper extremity (except hand)		
8. হাত Hand		
9. নিম্নাঙ্গ (পায়ের পাতা ব্যতীত) Lower extremity (except foot)		
10.পায়ের পাতা Foot		
11.কোমর Waist		

ইনজুরি ধরনের কোড Injury types:			
01. হাড় ভাঙ্গা	04. কেটে যাওয়া / উন্মুক্ত ক্ষত	07.পুড়ে যাওয়া	10. উরুতে আঘাত
02.মচকে যাওয়া	05. কামড় Bite	08.মাথায় আঘাত	97. অন্যান্য (উল্লেখ করুন)
03. হাড় সরে যাওয়া	06. ছিলে/ থেঁতলে যাওয়া	09. শরীরের ভিতরের অঙ্গের ক্ষত	

E 08.	যখন ইনজুরি ঘটেছিল তখন ব্যক্তিটির অবস্থা কেমন ছিল?	সজ্ঞানConscious…………………………………………......<br>সংজ্ঞাহীনUnconscious………………………………………..<br>জানা নেই (Unknown)………………………......................	01<br>02<br>99	 →E10<br>→E10
E 09.	যখন ইন্‌জুরি ঘটেছিল, তখন ব্যক্তিটির চলাফেরা করার ক্ষমতা কেমন ছিল? (যদি জ্ঞান থাকে)	একাএকা হাঁটতে পেরেছিলMobile alone………………………<br>অন্যের সহযোগিতায় হাঁটতে পেরেছিলMobile with assistance……….<br>হাঁটতে পারছিল নাImmobile…………………………………….<br>জানা নেইDon't know…………………………………………	01<br>02<br>03<br>99	
E 10.	আহত ব্যক্তিটি কি প্রাথমিক চিকিৎসা পেয়েছিল?	হ্যাঁYes…………………………………………...............<br>নাNo……………………………………………..................<br>জানা নেইDon't know…………………................................	01<br>02<br>99	Skip<br>→E16<br>→E16
E 11.	উত্তর হ্যাঁ হলে, কে প্রাথমিক চিকিৎসা দিয়েছিল ?	নিজ Himself/herself…………………………………….…<br>মা Mother……..<br>বাবা Father………..<br>স্বামী-স্ত্রী Husband/wife……………………………………………<br>ফার্মেসী/পল্লী চিকিৎসক Medicine shopkeeper/village doctor রেজিস্টার্ড Registered ……….<br>অন্য প্রাপ্ত বয়স্ক সেবাদানকারী Other adult caregiver………………….<br>ভাই/বোন Brother/sister……………………………………………<br>বন্ধু / সমবয়সী শিশু Friend/peer………………………………………<br>প্রতিবেশী Neighbor………..<br>মাঠ পর্যায়ের স্বাস্থ্যকর্মী Community health worker………………<br>কমিউনিটির স্বেচ্ছাসেবী কর্মী Community volunteer…………<br>ডাক্তার Doctor. ....<br>অন্যান্য (উল্লেখ করুন) Others (Specify)………………..…………….	01<br>02<br>03<br>04<br>05<br>06<br>07<br>08<br>09<br>10<br>11<br>12<br>13	
E 12.	সে কি প্রাথমিক চিকিৎসায় প্রশিক্ষণ প্রাপ্ত ?	হ্যাঁYes……………………………………………………..<br>নাNo……………………………………………………….<br>জানা নেইDon't know………………………………………	01<br>02<br>99	
E13.	ইনজুরির জন্য কোন চিকিৎসা গ্রহন করা হয়েছিল কি ?	হ্যাঁYes……………………………………………………..<br>নাNo…………………………………………..........................<br>জানা নেই Don't know	01<br>02<br>99	
E 14.	আহত ব্যক্তিকে কে চিকিৎসা করেছিল?	রেজিস্টার্ড ডাক্তার Registered doctor..…...<br>মেডিকেল এসিস্ট্যান্ট SACMO Medical Assistant/SACMO..….......<br>স্বাস্থ্য সহকারী/পরিবার কল্যাণ পরিদর্শিকা/পরিবার কল্যাণ সহকারী Health Assistant/Family Welfare Visitor/Family Welfare Assistant……<br>এন. জি. ও স্বাস্থ্য কর্মী NGO Service Provider……………………<br>ঔষধের দোকানদার/পল্লী চিকিৎসক……………………………………<br>Medicine shopkeeper/Village doctors<br>হোমিওপ্যাথিক চিকিৎসক Homeopathic Practitioner…………….<br>কবিরাজ/ ওঝা Herbal Medicine Practitioner………………<br>অন্যান্য উল্লেখ করুন Others (Specify) _____________ ___________	01<br>02<br>03<br>04<br>05<br><br>06<br>07<br>08	

E 15.	আহত ব্যক্তিটি কোথায় চিকিৎসা নিয়েছিল?	বিশেষায়িত হাসপাতাল মেডিকেল কলেজ হাসপাতাল, পঙ্গু হাসপাতাল, পি. জি. হাসপাতাল ইত্যাদি) ……………………………………………<br>Specialized hospital (Medical College Hospital, Orthopaedic Hospital, Post graduate Hospital etc)<br>জেলা হাসপাতাল District Hospital…………………………………...<br>উপজেলা স্বাস্থ্য কমপ্লেক্স Upazila Health Complex……………........<br>ইউনিয়ন স্বাস্থ্য ও পরিবার কল্যাণ কেন্দ্র ………………………………….<br>Union Health and Family Welfare Centre<br>প্রাইভেট ক্লিনিক Private Clinic…………………………………<br>কমিউনিটি ক্লিনিক Community clinic…………………………<br>এন.জি.ও ক্লিনিক NGO Clinic………………………………………<br>সেবাদানকারীর প্রাইভেট চেম্বার Private practitioners' chambers……<br>ফার্মেসী/ঔষধের দোকানদার…………………………………………..<br>Pharmacy/medicine shopkeeper<br>নিজ বাড়ীতে Own home……………………………………………...<br>অন্যান্য (উল্লেখ করুন) Others (Specify) ______________________	01<br><br><br>02<br>03<br>04<br><br>05<br>06<br>07<br>08<br>09<br><br>10<br>97	
E 16.	আহত ব্যক্তি হাসপাতালে/স্বাস্থ্যকেন্দ্রে ভর্তি হয়েছিলকি?	হ্যাঁYes……………...<br>না No……………………………………………........................<br>জানা নেই Don't know………………………………………………..	01<br>02<br>99	 → E25<br>→ E25
E 17.	(উত্তর'হ্যাঁ'হলে) আহত ব্যক্তিকে যদি হাসপাতাল/স্বাস্থ্যকেন্দ্র /ক্লিনিকে ভর্তি করা হয়ে থাকে তাহলে কোন ধরনের হাসপাতাল ?<br>?	বিশেষায়িত হাসপাতাল (মেডিকেল কলেজ হাসপাতাল, পঙ্গু হাসপাতাল, পি. জি. হাসপাতাল ইত্যাদি) …………………………<br>Specialized hospital (Medical College Hospital, Orthopaedic Hospital, Post graduate Hospital etc.)<br>জেলা হাসপাতাল District Hospital…………………………………...<br>উপজেলা স্বাস্থ্য কমপ্লেক্স Upazila Health Complex…………………<br>প্রাইভেট ক্লিনিক Private Clinic…………………………………<br>এন.জি.ও ক্লিনিক NGO Clinic……………………………………<br>অন্যান্য (উল্লেখ করুন) Others (Specify) ______________________	01<br><br>02<br>03<br>04<br>05<br>97	
E 18.	হাসপাতালে/চিকিৎসা প্রদানকারীর নিকট কিভাবে নিয়ে যাওয়া হয়েছিল ?	এ্যাম্বুলেন্স Ambulance……………………………………………..<br>অন্য কোন মোটর চালিত গাড়িতে (বাস, জীপ, কার এবং তিন চাকাবিশিষ্ট গাড়ি) Other<br>মোটরবিহীন যানবাহন (রিক্সা, রিক্সা ভ্যান, ঠেলাগাড়ি) ....<br>নৌকা Boat……………………………….............................<br>ফেরী /লঞ্চ /স্টীমার Ferry/launch/steamer……………………….......<br>অন্যান্য (উল্লেখ করুন) Others (Specify)_______________________<br>জানা নেই Don't know………………………………………………	01<br>02<br>03<br>04<br>05<br>97<br>99	
E 19.	আঘাত পাওয়ার পর হাসপাতালে/সেবাদানকারীর নিকট পৌছাতে কত সময় লেগেছিল ? (ঘন্টায় লিখুন) [যদি এক ঘন্টার কম হয় তবে 000লিখুন	|__|__|__|		
E 20.	ব্যক্তিটি হাসপাতালে কতদিন ভর্তি ছিল ?	|__|__|__|		

E 21.	আহত ব্যক্তির সার্জারী বা অপারেশন লেগেছিল কি?	হ্যাঁYes………………………………………………………..….<br>নাNo……………………………………………………………<br>জানা নেইDon't know……………………………………………	01<br>02<br>99	 →E23<br>→E23
E 22.	উত্তর'হ্যাঁ' হলে অপারেশনে কি ধরনের অ্যানেস্থেসিয়া দেয়া হয়েছিল?	স্থানীয়Local……………………………………………………<br>সম্পূর্ন General………………………………………………<br>দেয়া হয়নিNot given…………………………………………<br>জানা নেইDon't know……………………………………………	01<br>02<br>03<br>99	
E 23.	চিকিৎসার ফলাফল কি ছিল ?	সম্পূর্ণ সুস্থ হয়েগিয়েছে Recovered……………….….............<br>উন্নতিহয়েছে Improving……………………………….…………..…<br>কোন উন্নতি হয়নি No improvement ……………….……..………<br>অবনতি ঘটছে Deteriorating………………..................................	01<br>02<br>03<br>04	
E 24.	চিকিৎসার জন্য সর্বমোট কত টাকা খরচ হয়েছিল ?	পরামর্শ ফি Consultation fee… |__|__|__|__|__| ল্যাবরেটরি পরীক্ষা খরচ…………..… Laboratory investigation cost |__|__|__|__|__| বেড ফি Bed fee………………... |__|__|__|__|__| অপারেশন খরচ…………............ Operation cost |__|__|__|__|__| ঔষধপত্রMedicinecost……….......... |__|__|__|__|__| রোগীর সাহায্যকারীর থাকার খরচ . . . . Attendant's accommodation cost |__|__|__|__|__| যাতায়াত Transport cost……….. |__|__|__|__|__| অন্যান্য Others…………………... |__|__|__|__|__| সর্বমোট Total………………… |__|__|__|__|__|		
E 25.	ইন্জুরির ফলে কোন পঙ্গুত্ব হয়েছে কি?	হ্যাঁYes……………………………………….......................<br>নাNo……………………………………………………………..<br>জানা নেইDon't know……………..	01<br>02<br>99	 →E29<br>→E29
E 26.	উত্তর হ্যাঁ হলে, তা কি ধরনের পঙ্গুত্ব?	দৃষ্টি শক্তি নষ্ট হয়ে গেছে Loss of vision………………………………<br>শ্রবণ শক্তি নষ্ট হয়ে গেছে Loss of hearing…………….........................<br>হাঁটার শক্তি নষ্ট হয়ে গেছে Loss of mobility…………………………<br>হাত দিয়ে কাজ করার শক্তি নষ্ট হয়ে গেছে Loss of activity with hand………<br>বাক শক্তি নষ্ট হয়ে গেছে Loss of speech………………………………<br>বুদ্ধি শক্তি নষ্ট হয়ে গেছে Loss of intellect……………………………<br>অন্যান্য (উল্লেখ করুন) Others (Specify) ______________________	01<br>02<br>03<br>04<br>05<br>06<br>97	 <br><br><br>→E29<br>→ E 29<br>→ E 29
E 27.	এই ইনজুরি কি শরীরের কোন অঙ্গের এক/উভয় দিককেই পঙ্গু করেছিল ?	দৃষ্টি শক্তি নষ্ট হয়ে গেছে Loss of vision<br>এক দিকে Unilateral……………..………….....................................<br>দুই দিকে Bilateral……………………….....................................<br><br>শ্রবণ শক্তি নষ্টহয়ে গেছে Loss of hearing	 01<br>02	

		এক দিকেUnilateral……………………………………….………	01	
		দুই দিকেBilateral……………………………..............................	02	
		হাঁটার শক্তি নষ্টহয়ে গেছে Loss of mobility:		
		এক দিকেUnilateral……………………………………..	01	
		দুই দিকেBilateral………………………………………	02	
		হাত দিয়ে কাজ করার শক্তি নষ্ট হয়ে Loss of activity with hand		
		এক দিকে Unilateral……………………….……………	01	
		দুই দিকে Bilateral……………………..........................	02	
E 28.	অসুস্থতার কারনে কতদিন স্বাভাবিক কাজকর্মে অন্যের সাহায্য গ্রহন করতে হয়েছিল?<br>(দিনে লিখুন)	হাঁটা Walking ………………….….…… |__|__| |__| বসা এবং বিছানা থেকে উঠা…………… |__|__| |__| Sitting down and getting out of bed<br>সিঁড়ি বেয়ে উঠা নামা…..…………… |__|__| |__| Climbing stair<br>গোসলকরা…………………………..… . |__|__| |__| Bathing<br>টয়লেট ব্যবহার করা……………………… |__|__| |__| Using toilet		
E 29.	অসুস্থতার কারনে কতদিন স্বাভাবিক কাজকর্ম করতে পারেনি?	|__|__| |__|		
E 30.	ব্যক্তিটি কি আপনাদের পরিবারের একজন উল্লেখযোগ্য আয়-উপার্জনকারী ?	প্রধান Main …………………………………………….…	01	
		অন্যতম তবে প্রধান নয় Major but not main...	02	
		গৌণ Minor ………………………………………..................	03	→ END
		উপার্জনকারী নন None…………………………..…………………	04	→ END
		উত্তর দিতে সমর্থ নন Unable to answer…………………....................	05	→ END
E 31.	পরিবারটি এই উপার্জনের ক্ষতি কিভাবে পুষিয়ে নিয়েছিল?	আত্মীয়/বন্ধু/প্রতিবেশীর কাছ থেকে সুদমুক্ত ধার…………………	01	
		কোন ব্যক্তি বা সংস্থা থেকে সুদসহ ধার…………………………….	02	
		স্থাবর-অস্থাবর সম্পদ বিক্রিSell assets and household possession	03	
		খাবারের পরিমাণ কমিয়েReduce family consumption of food………	04	
		অন্যান্য প্রয়োজনীয় খরচ কমিয়ে (যেমন, কাপড়, আশ্রয়)…………….	05	
		অন্যান্য উল্লেখ করুন Others specify …………………………………	97	
		জানা নাই Don't know………………………………………………	99	

Module IV

Section F: Information on injury mortality

ইনজুরি জনিত মৃত্যু ফর্ম (Injury Mortality Form)

Household unique number	[ ] [ ][ ][ ][ ][ ][ ][ ][ ]
Person number	[ ][ ]
Person name:	

No.	Questions	Coding Categories		Skip
F 01.	মৃত ব্যক্তির লিঙ্গ<br>Sex of deceased person	পুরুষ Male……………………………………………………<br>মহিল ।Female……………………………………………………<br>হিজড়া (Transgender) ……………………………………………	1<br>2<br>3	
F 02	কিভাবে ইনজুরি হয়েছিল ?<br>What was the external cause of injury?<br><br>(নির্দিষ্ট ইনজুরি পদ্ধতি ফর্ম পূরণ করুন<br>Please fill in the specific injury mechanism form)	আত্মহত্যা-এম ১ Suicide - M1 ……………………………………<br>সড়ক দুর্ঘটনা-এম ২ Transport injury- M2……………………………<br>সহিংসতা -এম ৩ Violence-M3…………………………………………<br>পড়ে যাওয়া -এম ৪ Fall-M4…………………………………………………<br>ধারালো বস্তু দ্বারা কেটে যাওয়া-এম ৫ Cut injury-M5……………………<br>পুড়ে যাওয়া-এম ৬ Burn-M6……………………………………………...<br>ডুবেযাওয়া-এম ৭ Drowning-M7………………………………………<br>দুর্ঘটনাজনিত বিষপান-এম ৮ Unintentional poisoning -M8……………<br>মেশিন/যন্ত্রপাতির আঘাত -এম ৯ Machine injury-M9……………………<br>বিদ্যুৎস্পৃষ্ট -এম ১০ Electrocution-M10…………………………………<br>প্রানী ও কীট পতঙ্গের কামড় / আঘাত-এম ১১ Animal injury-M11………<br>ভোঁতা বস্তুর আঘাত -এম ১২ Injury byblunt object-M12……………..…<br>দুর্ঘটনাজনিত শ্বাসরোধ-এম ১৩ Suffocation-M13……………………<br>অন্যান্য (উল্লেখকরুন) Others (Specify)_________________________<br>জানা নেই Don't know………………………………………………	01<br>02<br>03<br>04<br>05<br>06<br>07<br>08<br>09<br>10<br>11<br>12<br>13<br>97<br>99	
F 03.	কত তারিখে ইনজুরি ঘটেছিল?<br>What was the date of injury?	|_D_|_D_| |_M_|_M_| |_Y_|_Y_|_Y_|_Y_|		
F 04.	কখন ইনজুরি ঘটেছিল? (২৪ঘন্টায় লিখুন) What was the time of injury? (in 24 hours)	|_H_|_H_| |_M_|_M_|		
F 05.	ইনজুরি ঘটার সময় ব্যক্তিটি কোথায় ছিল?<br>Where was the person when s/he was injured?	শোয়ার ঘর Bedroom………………………………………………<br>বসার ঘর Living room……………………………………………<br>রান্না ঘর Kitchen…………………………………………………<br>গোসল খানা/পায়খানা Bathroom…………………………………<br>বাড়ীর উঠান Yard………………………………………………......<br>বারান্দা Veranda...<br>এক কক্ষ বিশিষ্ট ঘর One room dwelling ……………………………<br>শ্রেনীকক্ষ Classroom...<br>স্কুলের খেলার মাঠ School playground…………………………......<br>শিক্ষা প্রতিষ্ঠানের হোস্টেল Hostel of educational institute………………<br>অন্য খেলাধুলার স্থান/ ক্রীড়াঙ্গন Other playground………………………<br>রাস্তায় /মহাসড়কে Roads/highway……………………………………<br>রেল ষ্টেশন/ফেরী/লঞ্চ ঘাট/বাসস্ট্যান্ড/অন্য পরিবহনএলাকা Railway station/Ferry/ Launch station/Bus stand/ Other vehicles area…………	01<br>02<br>03<br>04<br>05<br>06<br>07<br>08<br>09<br>10<br>11<br>12<br>13	

		কৃষিক্ষেত্র/খামার বাড়ী/চাতাল Agricultural field …………………………<br>শিল্প/কলকারখানা/ওয়ার্কশপ Industry/factory/workshop…………………<br>জলাশয় Water reservoir………………………………………………………<br>হাট/ বাজার Market/haat/bazaar…………………………………………<br>অফিস Office………………………………………………………………<br>নির্মাণ এলাকা Construction area………………………………………<br>অন্যান্য (উল্লেখ করুন) Others (Specify)__________________________<br>জানা নেই Don't know………………………………………………….	14<br>15<br>16<br>17<br>18<br>19<br>97<br>99	
F 06.	ইন্জুরির অভিপ্রায় কি ছিল?<br>What was the intent of the injury?	অনিচ্ছাকৃত Unintentional…………………………………………<br>ইচ্ছাকৃত নিজের ক্ষতি Intentional/Self harm…………………………<br>সহিংসতা Assault/Violence…………………………………………......<br>নির্ণয় করা যায়নি Undetermined……………………………………	01<br>02<br>03<br>04	

E 07. ব্যক্তিটির শরীরের কোন কোন অঙ্গে এবংকি ধরনের ইনজুরি হয়েছিল?		
শরীরের কোন অঙ্গে	ক্ষত অঙ্গ	ইনজুরি ধরনের কোড
1. মাথা Head		
2. মুখমন্ডল Face		
3. চোখ Eye		
4. ঘাড় Neck		
5. বুক/পিঠ Chest		
6. পেট Abdomen		
7. বাহু হাত ব্যতীত Upper extremity (except hand)		
8. হাত Hand		
9. নিম্নাঙ্গ (পায়ের পাতা ব্যতীত) Lower extremity (except foot)		
10.পায়ের পাতা Foot		
11.কোমর Waist		

ইনজুরি ধরনের কোড Injury types:			
01. হাড় ভাঙ্গা	04. কেটে যাওয়া / উন্মুক্ত ক্ষত	07.পুড়ে যাওয়া	10. উরুতে আঘাত
02.মচকে যাওয়া	05. কামড় Bite	08.মাথায় আঘাত	97. অন্যান্য (উল্লেখ করুন)
03. হাড় সরে যাওয়া	06. ছিলে/ থেঁতলে যাওয়া	09. শরীরের ভিতরের অঙ্গের ক্ষত	

F 08.	যখন ইনজুরি ঘটেছিল তখন ব্যক্তিটির অবস্থা কেমন ছিল? What was the condition of the victim just after injury?	সজ্ঞান<br>Conscious……………………………..……………………........<br>সংজ্ঞাহীন Unconscious……………………………….………………<br>জানা নেই Unknown……………………………..............................	01<br>02<br>99	
F 09.	যখন ইনজুরি ঘটেছিল, তখন ব্যক্তিটির চলাফেরা করার ক্ষমতা কেমন ছিল? (যদি জ্ঞান থাকে)<br>What was the mobility condition of the person just after injury (if answer is conscious)	একা একা হাঁটতে পেরেছিল Mobile alone……………………………<br>অন্যের সহযোগিতায় হাঁটতে পেরেছিল Mobile with assistance………<br>হাঁটতেপারছিলনা Immobile…………………………………………<br>জানা নেই Don't know…………………………………………………	01<br>02<br>03<br>99	
F 10.	ব্যক্তিটি কি প্রাথমিক চিকিৎসা পেয়েছিল?<br>Did the person receive first aid?	হ্যাঁYes……………………………………………………………..<br>নাNo………………………………………………………………<br>জানা নেই Don't know…………………………………………………	01<br>02<br>99	
F 11.	উত্তর হ্যাঁ হলে, কে প্রাথমিক চিকিৎসা দিয়েছিল?<br>If yes, who gave the person first aid?	নিজেই Himself/herselfOwn…………………………………………01<br>মা Mother………………………………………………….............02<br>বাবাFather ……………………………………………………… 03<br>স্বামী/স্ত্রী Husband/wife…………………………………………… 04<br>ফার্মেসী/পল্লী চিকিৎসক Medicine shopkeeper/village doctor……… 05<br>অন্য প্রাপ্ত বয়স্ক সেবাদানকারী Other adult caregiver…………………… 06<br>ভাই/বোন Brother/sister…………………………………………...... 07<br>বন্ধু / সমবয়সী শিশু Friend/peer……………………………………… 08<br>প্রতিবেশী Neighbor………………………………………………… 09<br>মাঠ পর্যায়ের স্বাস্থ্যকর্মী Community health worker………………… 10<br>কমিউনিটির স্বেচ্ছাসেবী Community volunteer…………………… 11<br>ডাক্তার Doctor …………………………………………………… 12<br>অন্যান্য (উল্লেখ করুন) Others (specify) ________________________ 97		
F 12.	সে কি প্রাথমিক চিকিৎসায় প্রশিক্ষণপ্রাপ্ত ?<br>Was s/he trained in first aid?	হ্যাঁ Yes………………………………………………………........<br>না No………………………………………………………………<br>জানা নেই Don't known……………………………………………………	01<br>02<br>99	
F 13.	ইনজুরির জন্য কোন চিকিৎসা গ্রহন করা হয়েছিল কি ?<br>Did the person receive treatment for injury?	হ্যাঁ Yes…………………………………………………………........<br>না No………………………………………………………………<br>জানা নেই Don't know…………………………………………………	01<br>02<br>99	
F 14.	আহত ব্যক্তিকে কে চিকিৎসা দিয়েছিল?<br>Who provided the treatment?	রেজিষ্টার্ড ডাক্তার Registered doctor..…..<br>মেডিকেল এসিস্ট্যান্ট SACMO Medical Assistant/SACMO..…........<br>স্বাস্থ্য সহকারী/পরিবার কল্যাণ পরিদর্শিকা/পরিবার কল্যাণ সহকারী Health Assistant/Family Welfare Visitor/Family Welfare Assistant………..<br>এন. জি. ও স্বাস্থ্য কর্মী NGO Service Provider……………………<br>ঔষধের দোকানদার/পল্লী চিকিৎসক……………………………………<br>Medicine shopkeeper/Village doctors<br>হোমিওপ্যাথিক চিকিৎসক Homeopathic Practitioner…………….<br>কবিরাজ/ওঝা Herbal Medicine Practitioner………………<br>অন্যান্য উল্লেখ করুন Others (Specify) _____________ ___________	01<br>02<br>03<br>04<br>05<br>06<br>07<br>97	
F 15.	ব্যক্তিটি কোথায় চিকিৎসা নিয়েছিল?<br><br>Where did the person receive treatment?	বিশেষায়িত হাসপাতাল (মেডিকেল কলেজ হাসপাতাল, পঙ্গু হাসপাতাল, পি. জি. …… হাসপাতাল ইত্যাদি)<br>জেলা হাসপাতাল District Hospital………………………………<br>উপজেলা স্বাস্থ্য কমপ্লেক্স Upazila Health Complex………………………<br>ইউনিয়ন স্বাস্থ্য ও পরিবারকল্যাণ কেন্দ্র …………………………………<br>প্রাইভেট ক্লিনিক Private Clinic……………………………………<br>এন.জি.ও ক্লিনিক NGO Clinic………………………………………..	01<br>02<br>03<br>04<br>05<br>06	

		সেবাদানকারীর প্রাইভেট চেম্বার Private practitioners' chambers……<br>ফার্মেসী/ঔষধের দোকানদার Pharmacy/medicine shop…………………<br>নিজবাড়ী Own home………………………………………………………<br>অন্যান্য (উল্লেখ করুন) Others (Specify)________________________	07<br>08<br>09<br>97	
F 16.	ব্যক্তিটি হাসপাতালে/স্বাস্থ্যকেন্দ্রে ভর্তি হয়েছিল কি ?<br>Was the person admitted to a health facility?	হ্যাঁ Yes……………………………………………………………......<br>না No…………………………………………………………………<br>জানা নেই Don't know………………………………………………	01<br>02<br>99	
F 17.	(উত্তর হ্যাঁ হলে) ব্যক্তিটি যদি হাসপাতাল / স্বাস্থ্যকেন্দ্র /ক্লিনিকে ভর্তি হয়ে থাকে তাহলে কোন ধরনের হাসপাতালে ?<br>If the person was admitted, what type of health facility was s/he admitted to?	বিশেষায়িত হাসপাতাল (মেডিকেল কলেজ হাসপাতাল, পঙ্গু হাসপাতাল, পি. জি. হাসপাতাল ইত্যাদি)<br>জেলা হাসপাতাল District Hospital…………………………………………<br>উপজেলা স্বাস্থ্য কমপ্লেক্স Upazila Health Complex…..…………………<br>প্রাইভেট ক্লিনিক Private Clinic……………………………………<br>এন.জি.ও ক্লিনিক NGO Clinic………………………………………<br>অন্যান্য (উল্লেখ করুন) Others (Specify)________________________	01<br>02<br>03<br>04<br>05<br>97	
F 18.	ব্যক্তিটিকে হাসপাতালে/সেবাদানকারীর নিকট কিভাবে নিয়ে যাওয়া হয়েছিল?<br>How was the patient transported to the health facility?	এম্বুলেন্স Ambulance……………………………………………………<br>অন্য কোন মোটর চালিত গাড়িতে (বাস, জীপ, কার এবং তিন চাকাবিশিষ্ট গাড়ি<br>মোটরহীন যানবাহন (রিকশা, রিকশা ভ্যান, ঠেলাগাড়ি)<br>নৌকা Boat……………………………………………………..…<br>ফেরী /লঞ্চ /স্টীমার Ferry/launch/steamer……………….….….<br>অন্যান্য (উল্লেখ করুন) Others (Specify) ________________________<br>জানা নেই Don't know…………………………………………..…	01<br>02<br>03<br>04<br>05<br>97<br>99	
F 19.	আঘাত পাওয়ার পর হাসপাতালে/সেবাদানকারীর নিকট পৌছাতে কত সময় লেগেছিল ? (ঘন্টায় লিখুন) [যদি এক ঘন্টার কম হয় তবে 000 লিখুন]	|___|___|___|		
F 20.	ব্যক্তি হাসপাতালে কতদিন ভর্তি ছিল ?	|___|___|___|		
F 21.	সার্জারী বা অপারেশন হয়েছিল কি ?	হ্যাঁ Yes……………………………………………………………............<br>না No…………………………………………………………………<br>জানা নেই Don't know………………………………………………	01<br>02<br>99	
F 22.	যদি হ্যাঁ হয়, তাহলে কি ধরনের অ্যানেস্থেসিয়া দেয়া হয়েছিল?	স্থানীয় Local……………………………………………………………<br>সম্পুর্ন General……………………………………………………........<br>দেয়া হয় নি Not given………………………………………………<br>জানা নেই Don't know………………………………………………	01<br>02<br>03<br>99	
F 23.	চিকিৎসার জন্য সর্বমোট কত টাকা খরচ হয়েছিল?	পরামর্শ ফি Consultation fee… |___|___|___|___|___| ল্যাবরেটরি পরীক্ষা খরচ…..… Laboratory investigation cost |___|___|___|___|___| বেডফি Bed fee……………….... |___|___|___|___|___| অপারেশন খরচ…………….............. Operation cost |___|___|___|___|___| ঔষধপত্র Medicine ost……….......... |___|___|___|___|___| রোগীর সাহায্যকারীর থাকার খরচ… Attendant's accommodation cost |___|___|___|___|___| যাতায়াত Transport cost………… |___|___|___|___|___| অন্যান্য Others………………….... |___|___|___|___|___|		

		সর্বমোট Total………………… |___|___|___|___|___|		
F 24.	ব্যক্তিটির (নাম উল্লেখ করুন) কবে মৃত্যু হয়েছিল?	|D|D| |M|M| |Y|Y|Y|Y|		
F 25.	ব্যক্তিটির (নাম উল্লেখ করুন) কখন মৃত্যু হয়েছিল? (২৪ ঘন্টায় লিখুন)	|___|___| |___|		
F 26.	ব্যক্তিটির (নাম উল্লেখ করুন) কি ঘটনাস্থলেই মৃত্যু হয়েছিল?	হ্যাঁ Yes…………………………………………………………………..<br>না No……………………………………………………………………..<br>জানা নেই Don't know…………………………………………………	01<br>02<br>99	
F 27.	কোন জায়গায় মারা গিয়েছিল?	হাসপাতালে Hospital…………………………………………….…<br>নিজবাড়ীতে Home……………………………………………………..<br>রাস্তায় On the road…………………………………………………..…<br>বাড়ীতে নেওয়ার পথে Way to home……………………..……………<br>হাসপাতালে নেওয়ার পথে Way to hospital…………………………….……<br>অন্যান্য (উল্লেখ করুন)Others (Specify)……………….…….…………	01<br>02<br>03<br>04<br>05<br>97	
F 28.	মৃত ব্যক্তির ময়না তদন্ত করা হয়েছিল কি?	হ্যাঁYes……………………………………………………………………<br>নাNo………………………………………………………………………..<br>জানা নেইDon't know…………………………………………………..…	01<br>02<br>99	
F 29.	ব্যক্তিটি কি আপনাদের পরিবারের একজন উল্লেখযোগ্য আয় উপার্জনকারী ?	প্রধান Main ……………………………………………………………...<br>অন্যতম কিন্তু প্রধান নয় Major but not main……………………………..<br>গৌণ Minor………………………………………………………………<br>উপার্জনকারী নন None…………………………………………………..<br>জানা নেই Don't know…………………………………………………...	01<br>02<br>03<br>04<br>99	 → I31<br><br>→ I31<br>→ I31
F 30.	পরিবারটি এই উপার্জনের ক্ষতি কিভাবে পুষিয়ে নিয়েছিল ?	আত্মীয়/বন্ধু/প্রতিবেশী কাছ থেকে সুদমুক্ত ধার……………………… Borrow money from relatives/neighbor/friends<br>কোন ব্যক্তি বা সংস্থা থেকে সুদসহ ধার……………………………… Took loans from person /institutions/organization<br>স্থাবর-অস্থাবর সম্পদ বিক্রি Sell assets and household possession…….<br>খাবারের পরিমাণ কমিয়েReduce family consumption of food…..……..…<br>অন্যান্য প্রয়োজনীয় খরচ কমিয়ে (যেমন, কাপড়, আশ্রয়)…………….…… Decrease expenditure on other basic needs, e.g. clothing and shelter<br>অন্যান্য উল্লেখকরুন Others specify ……………………………..………<br>জানা নাই Don't know…………………………………………….……	01<br>02<br>03<br>04<br>05<br>97<br>99	
F 31.	মৃত্যুর ক্ষেত্রে ঘটনার সংক্ষিপ্ত বিবরণ	………………………………………………………………………<br>………………………………………………………………………<br>…………………………………………….……….…………		→ END

Module V (M1) Section G: এম ১: আত্মহত্যার প্রচেষ্টা/ আত্মহত্যা

House hold identification number

Person Number

Person Name:

No.	Questions	Coding Categories		Skip
G01.	কোথায় আত্মহত্যা চেষ্টা/ আত্মহত্যা করেছিল?	নিজবাড়ী Own house………………………………………...	01	
		শ্বশুরবাড়ী In laws house……………………………........	02	
		অন্যেরবাড়ী Other's house…………………………........	03	
		সড়ক/জনপথ Highway/street……………………………........	04	
		জলাশয় Water body……………………………………......	05	
		গাছ Tree…………………………………………………	06	
		রেল লাইন Rail Line…………………..………………………	07	
		অন্যান্য (উল্লেখ করুন) Others (Specify)______________	99	
G02.	কিভাবে আত্মহত্যার চেষ্টা/ আত্মহত্যা করেছিল?	বিষ পান Poisoning….……………………………………..	01	
		ফাঁসি Hanging………………………………………….......	02	
		পানিতে ডুবা Drowning…..………………………………..	03	
		পুড়ে যাওয়া Burn…………………………………………….........	04	
		রক্তক্ষরণExsanguination………………………………….......	05	
		উপর থেকে লাফ দেওয়া Jump from height…………………......	06	
		গুলিবিদ্ধ হওয়া Gunshot………………………………………	07	
		ঘুমের বড়ি খাওয়ার মাধ্যমে……………………………………	08	STOP
		অন্যান্য (উল্লেখ করুন) Others (Specify) _________________	97	→
		জানা নেই Don't know………………………………………	99	
G03.	যদি বিষপান হয়, তাহলে বিষের প্রকার কি ছিল?	কীটনাশক Pesticides………………………………………	01	
		মশা, মাছি, তেলাপোকা ইত্যাদি ধ্বংসকারী Insecticides……	02	
		ইদুঁর মারা বিষ Rodenticides………………………………	03	
		ঘুমের বড়ি Sleeping pills………………………………	04	
		অন্যান্য ঔষধ Other medicine…………………………	05	
		সাবান, গুঁড়া সাবান Soap/detergent…………………………	06	
		কেরোসিন Kerosene……………………………………….	07	
		স্যাভলন / ডেঁল Savlon/Dettol……………………………	08	
		হারপিক Harpic……………………………………………	09	→END
		ব্লিচিং পাউডার Bleaching power……………………………….	10	
		চুন (Lime)………………………………………….	11	
		এসিড Acid…………………………………………………	12	
		অন্যান্য (উল্লেখ করুন)Others (Specify) _______________	97	
		জানা নেই Don't know …………………………………	99	

Module V (M2) Section H এম-২: পরিবহন দুর্ঘটনা

House hold identification number: [ ] [ ][ ][ ][ ][ ][ ][ ][ ]

Person Number: [ ][ ]

Person Name:

No.	Questions	Coding Categories		Skip
H 01.	দুর্ঘটনার সময় ব্যক্তিটি কিভাবে ভ্রমন করছিল?<br>How was the person travelling prior to the injury?	পায়ে হেঁটে Pedestrian…………………………………..……	01	
		সাইকেল Bicycle……………………………………………..	02	
		রিক্সা/ভ্যানRickshaw/Van……………………………………..	03	
		মটরসাইকেলMotorcycle…………………………………...	04	
		মটরকার Car…………………………………………………	05	
		অটো-রিক্সা /টেম্পু/পিক-আপ/ জিপগাড়ী/মাইক্রোবাস……...................... Auto-rickshaw/ Tempo/pick-up/Jeep/Microbus	06	
		ইসিমন Nosimon……………………………………………..	07	
		ট্রাক Truck………………………………………………	08	
		বাস/ মিনিবাস Bus /Mini bus…………………………	09	
		ট্রেন Train…………………………………………………	10	
		নৌকা Boat………………………………………………	11	
		ফেরী Ferry………………………………………………	12	
		স্টীমার/লঞ্চ Steamer /Launch…………………………	13	
		স্পীড বোট Speed boat………………………………………	14	
		গরু মহিষের গাড়ী …………………………………	15	
		ঘোড়া………………………………………………	16	
		অন্যান্য (উল্লেখ করুন) Others (Specify)………………………	97	
		জানা নেই Don’t know…………………………………….	99	
H 02.	ব্যক্তিটি রাস্তায় কি করছিল?<br>What was the injured person doing on the road?	হাঁটছিল Pedestrian……………………………………	01	
		গাড়ী চালাচ্ছিলDriver…………………………………	02	
		সাইকেল চালাচ্ছিলBicyclists…………………………….	03	
		মটরসাইকেল চালাচ্ছিলMotorcyclists………………………	04	
		হেলপার/কন্ট্রাক্টর Helper/conductor…………………………	05	
		যাত্রী Passenger………………………………………………	06	
		রাস্তায় কাজ করছিল **Working in the road** ………………………	07	
		রাস্তায় খেলা করছিল Playing in the road……………………	08	
		অন্যান্য (উল্লেখকরুন) Others (Specify)_________________	97	
		জানা নেইDon’t know……………………………………...	99	
H 03.	কিসের সাথে আহত ব্যক্তির বাতারবাহনেরসংঘর্ষ হয়?<br>With what did the injured person (or his vehicle) collide?	কোন সংঘর্ষ হয়নি No collision……………………………	01	
		সাইকেল Bicycle…………………………………………….	02	
		রিক্সা/ভ্যান Rickshaw/Van………………………………….	03	
		মটরসাইকেল Motorcycle……………………………......	04	
		মটরকার Car……………………………………………….	05	
		অটো-রিক্সা/টেম্পু/পিক-আপ/ জিপগাড়ী/মাইক্রোবাস……	06	
		ইসিমন Nosimon…………………………………………….	07	

		ট্রাক Truck……………………………………………<br>বাস/মিনিবাসBus /Mini bus………………………………<br>ট্রেনTrain……………………………………………………<br>নৌকা Boat…………………………………………………<br>ফেরীFerry……………………………………………………<br>স্টীমার/লঞ্চ Steamer /Launch…………………………………<br>পথচারী Pedestrian……………………………………………<br>স্থির বস্তু (দেয়াল, গাছ, খুঁটি ইত্যাদি) ……………………<br>অন্যান্য (উল্লেখ করুন) Others (Specify)__________________<br>জানা নেই (Don't know)…………………………………………	08<br>09<br>10<br>11<br>12<br>13<br>14<br>15<br>97<br>99	
H 04.	ব্যক্তিটি রাস্তায় কেন গিয়েছিল?<br>Why was the injured victim on the road?	স্কুলে যাচ্ছিল/ফিরছিল Way to school……………………………<br>কর্মস্থলে যাচ্ছিলেন/ফিরছিলেন Way to workplace…………<br>বাহনটি চালাচ্ছিলেন Driving…………………………………<br>রাস্তায় খেলা করছিল Playing……………………………………<br>চালককে সহযোগিতা করছিল Helping driver………………<br>বেড়াতে বেরিয়েছিল Wandering………………………………<br>রাস্তায়কাজকরছিল Working on road…………………………<br>অন্যান্য (উল্লেখ করুন) Others (Specify) ______________<br>জানা নেই Don't know………………………………	01<br>02<br>03<br>04<br>05<br>06<br>07<br>97<br>99	
H 05.	ব্যক্তিটি কি নিরাপত্তার জন্য কোন কিছু ব্যবহার করেছিল?<br>Did the person use any safety device?	হ্যাঁ Yes……………………………………………<br>না No………………………………………………<br>জানানেই Don't know…………………………………	01<br>02<br>99	 →H07<br>→H07
H 06.	যদি হ্যাঁ হয়, তাহলে কি ব্যবহার করেছিল?<br>If yes, what was the safety device?	বেল্ট S eat belt…………………………………………………<br>হ্যালমেট Helmet…………………………………………………<br>লাইফ জ্যাকেট Life Jacket ……………………………………<br>অন্যান্য (উল্লেখ করুন) Others (Specify) ________________<br>জানা নেই Don't know…………………………………………	01<br>02<br>03<br>97<br>99	
H 07.	রাস্তার অবস্থা কেমন ছিল?<br>What was the condition of the road?	ভাল অবস্থা Good condition ………………………<br>ত্রুটিপূর্ণ রাস্তার নক্সা Poor/faulty road design………………<br>ভাঙ্গাচোরা রাস্তা Poor road condition ………………………<br>দেখার অসুবিধা (ঝড়-বৃষ্টি, কুয়াশ, কম আলো - ভোর/সন্ধ্যা, কোন কিছুর বাধা<br>অন্যান্য (উল্লেখ করুন) Others (Specify) ________________<br>জানা নেইDon't know…………………………………………	01<br>02<br>03<br>04<br>97<br>99	
H 08.	ব্যক্তিটি কি মাদকদ্রব্য সেবন করেছিল?<br>Was the person under the influence of alcohol or other mood-altering substance?	হ্যাঁYes………………………………………………………<br>না No…………………………………………………………<br>জানানেই Don't know………………………………………	01<br>02<br>99	
H 08	চালক কি মোবাইল ফোনে কথা বলছিল?<br>The driver talking over mobile	হ্যাঁYes………………………………………………………<br>না No…………………………………………………………<br>জানানেই Don't know………………………………………	01<br>02<br>99	 <br>→END

Module V (M3) (Section I) : এম ৩: সহিংসতার তথ্য

House hold identification number

Person Number:

Person Name:

No.	Questions	Coding Categories		Skip
I 01.	কি কারণে সহিংসতা ঘটেছিল?	ঝগড়া/মারামারিQuarrel/fight………………………………	01	
		ডাকাতি/ছিনতাই Burglary or robbery……………………	02	
		সংঘবদ্ধ সহিংসতা/ সন্ত্রাসCommitting a crime (other than above)………………………………………………………	03	
		অন্যান্য (উল্লেখ করুন) Others (Specify___________________	97	
		জানা নেই Don't know……………………………………...	99	
I 02.	আক্রমনকারীর সাথে ঘটনার শিকার ব্যক্তির সম্পর্ক কি?	স্বামী/স্ত্রী Spouse……………………………………………	01	
		বাবা Father……………………………………………	02	
		মা Mother………………………………………………	03	
		সন্তান Son/daughter…………………………………………	04	
		ভাই/বোন Brother/sister……………………………………	05	
		দাদা/দাদী/ নানা/নানী (Grandparents)…………………………	06	
		অন্যান্য আত্মীয় (উল্লেখ করুন)Other relatives (specify)………	07	
		বন্ধু/সমবয়সী Friend/acquaintance……………………………	08	
		সহকর্মী Colleague……………………………………………	09	
		পরিচিত (অনাত্মীয়) Known (Non relatives)……………………	10	
		অপরিচিত Stranger……………………………………………		
		অন্যান্য (উল্লেখ করুন) Others (Specify) _____________ _____	97	
		জানা নেইDon't know…………………………………………	99	
I 03.	আক্রমণকারীর লিঙ্গ কি?	পুরুষ Male………………………………………………	1	
		মহিলা Female……………………………………………	2	
		হিজড়া (transgender) ………………………………………	3	
I 04.	কি দিয়ে আঘাত করেছিল?	লাঠি Stick/Club……………………………………………	01	
		ছুরি / ধারালো অস্ত্র Knife/cutting/tool………………………	02	
		আগুন Fire………………………………………………	03	
		বন্দুক/ অন্য আগ্নেয়াস্ত্র Gun/firearm………………………	04	
		এসিড Acid……………………………………………	05	
		শরীরের অংশ যেমন মুষ্টি, পা Person, including parts of the body (e.g. fists, feet)……………………………………………	06	
		বল্লম/টেটা.Bollom/Teta………………………	07	
		অন্যান্য (উল্লেখ করুন) Others (Specify) _____________ __	97	→ END
		জানা নেই Don't know……………………………………..	99	

Module V (M4) Section J: এম ৪-পড়ে যাওয়া

House hold identification number	☐ ☐☐☐☐☐☐☐☐
Person Number:	☐☐
Person Name:	

No.	Questions	Coding Categories		skip
J 01.	কোথা থেকে পড়েছিল?	একই স্তর থেকে Same level………………………………….	01	
		উঁচু স্তর থেকে Different level………………………………	02 →	J 04
		সমতল থেকে নিচে Different level ……………………..…	03 →	J 04
J 02.	কিভাবেএকই স্তর থেকে পড়ে গিয়েছিল?	হোঁচট খেয়ে Stumbled…………………………………….....	01	
		পিছলে বা কোন কিছুতে পা আঁটকে গিয়ে Slipped, tripped…………..	02	
		অন্য কারো ধাক্কা খেয়ে Was Pushed………………….	03	
		অন্যান্য (উল্লেখ করুন) Others (Specify) _____________ ____	97	
		জানা নেই Don't know………………………………………	99	
J 03.	একই স্তর থেকে পড়ে যাওয়ার স্থানটি কোথায় ছিল ?	গোসলখানা / পায়খানা Bathroom..…………………………	01	
		শোয়ার ঘর Bedroom……..…………………………………...	02	
		রান্না ঘর Kitchen……………..…………………………	03	
		উঠান Sidewalk……………..…………………………….....	04	
		রাস্তা Street…………………..………………………………	05	
		কর্মস্থল Workplace…………..………………………………	06	
		খেলাধূলার মাঠ Sports area……..………………………….....	07	
		অন্যান্য (উল্লেখ করুন) Others (Specify_______________	97 →	Stop
		জানা নেই Don't know………..……………………………	99 →	Stop
J 04.	কিভাবে উঁচু স্তর থেকে পড়ে গিয়েছিল?	দূর্ঘটনাক্রমে পড়ে গিয়ে Unintentional fall……………...	01	
		লাফ দিয়ে Jump…………………………………………….	02	
		অন্য কারো সাথে ধাক্কা খেয়ে Was Pushed………………	03	
		অন্যান্য (উল্লেখ করুন) Others (Specify)_____________ _	97	
		জানা নেই Don't know……………………………………	99	
J 05.	কোন উঁচু স্তর থেকে পড়ে গিয়েছিল ?	সিঁড়ি Stairs……………………………………………..	01	
		গাছ Tree………………………………………………....	02	
		বাড়ীর ছাদ Roof……………………………………………..	03	
		আসবাবপত্র Furniture…………………………………………	04	
		মই Ladder…………………………………………………..	05	
		কোন প্রাণীর পিঠ Back of animal………………………..	06	
		অন্যান্য (উল্লেখ করুন) Others (Specify)_____________ ____	97	
		জানা নেইDon't know………………………………………..	99	
J 06	সমতল থেকে নিচে পরে গেলে কোথায় ?	মাটির গর্তে ……………………………………………..…	01	
		জলাশয়ে ………………………………………………………	02	
		নদী /খালের পার হতে………………………….……………	03	
		পানির ড্রেনে …………………….…………………………	04	END
		পানির সেফটি ট্যাংক ……………………………………………	05	

Module V (M5) এম ৫-ধারালো বস্তুর আঘাত

House hold identification number: ☐ ☐☐☐☐☐☐☐☐

Person Number: ☐☐

Person Name:

Section K: Information on cut injury

No.	Questions	Coding Categories		Skip
K 01.	ধারালো বস্তুটি কি ছিল ?<br><br>What was the sharp object that cut the person?	ছুরি Knife………………………………………….<br>দা/বটি Boti/Da………………………………………<br>কাস্তে Sickle…………………………………………….<br>কাঁচি Scissors…………………………………………..<br>কোদাল Spade………………………………………..…..<br>টিন Tin………………………………………………..<br>শাবল/খুন্তি ……………………………….……..…..…..<br>শামুক ………………………………..……...……….<br>লাঙ্গলের ফলা……………………………………………..<br>পেরেক/তারকাটা………………………………………….<br>ব্লেড Bleade ……………………………………………<br>গাছের কাটা……………………………………………..<br>কাঁচভাঙ্গা Broken glass……………………………..….<br>অন্যান্য (উল্লেখ করুন) Others (Specify)______________	01<br>02<br>03<br>04<br>05<br>06<br>07<br>08<br>09<br>10<br>11<br>12<br>13<br>97	
K 02.	ব্যক্তিটি ঐ বস্তু দ্বারা কিভাবে আঘাত প্রাপ্ত হয়েছিল ?<br>How did the person get injured with the object?	কৃষিকাজ/কৃষি যন্ত্র দিয়ে কাজ করার সময়……………………..<br>ঘরের কাজ করার সময়…………………………………..…...<br>অন্যদের দ্বারা (অনিচ্ছাকৃত)……………………………..….<br>খেলা করার সময়………………………………………..….<br>অন্য কোন কাজ করার সময়……………………………….<br>বস্তুটিরওপরে পড়ে গিয়েছিল Fell on the object……………<br>অন্যান্য (উল্লেখ করুন) Others (specify)…………………	01<br>02<br>03<br>04<br>05<br>06<br>97	
K 03.	সাধারণতঃ কাজ শেষে ধারালো বস্তুটি কোথায় রাখা হয় ?<br>Where is the sharp objectusually stored?	রান্নাঘর Kitchen……………………………..<br>শোয়ার ঘর Bedroom……………………….……..<br>খাওয়ার ঘর Dining area…………………………<br>বসার ঘর Living area……………………………<br>গোসলখানা/পায়খানা Bathroom…………………<br>স্টোর রুম/গুদাম ঘর Storage room………………..<br>এক কক্ষ বিশিষ্ট ঘর Single room dwelling……………<br>বারান্দা Veranda……………………..<br>ঘরের বাহিরে……………………………..<br>অন্যান্য (উল্লেখ করুন) Others (Specify) ____________	01<br>02<br>03<br>04<br>05<br>06<br>07<br>08<br>09<br>97	END

Module V (M6) Section L: এম ৬-পুড়ে যাওয়া

House hold identification number: ☐ ☐☐☐☐☐☐☐☐

Person Number: ☐☐

Person Name:

No.	Questions	Coding Categories		Skip
L 01.	কি দ্বারা ব্যক্তিটি পুড়ে গিয়েছিল ?	অগ্নিশিখা Flame…………………………………………<br>উত্তপ্ত তরল Hot liquid……………………………….....<br>উত্তপ্ত বস্তু Hot object………………………………….<br>বিস্ফোরক Explosive…………………………………..<br>রাসায়নিক পদার্থ Chemical…………………………………<br>অন্যান্য (উল্লেখ করুন) Others (Specify)________________	01<br>02<br>03<br>04<br>05<br>97	 →L03<br>→L04<br>→L05<br>→L06<br>→L07
L 02.	যদি অগ্নি শিখা হয়, অগ্নি শিখার উৎস কি ছিল ?	রান্নার আগুন Cooking fire(wood, Leaf, cowdung)………<br>গ্যাসের আগুন Gas fire...<br>আগুন পোহানো Heating fire………………………………<br>কর্মস্থলে আগুন লেগে Work-place source……………………<br>বাড়ীতে আগুন লেগেResidential or house fire………………<br>কুপি/হারিকেন Kerosene lamp……………………………<br>বিদ্যুৎ Electrical burn…………………………………<br>মোমের আগুন Candle Fire……………………………….<br>অন্যান্য (উল্লেখ করুন) Others (Specify)________________	01<br>02<br>03<br>04<br>05<br>06<br>07<br>08<br>97	} L07
L 03.	যদি উত্তপ্ত তরল হয়, তরল পদার্থটি কি ছিল ?	রান্নার পানি Cooking water……………………………..…<br>গোসলের পানিBathing water………………………………<br>ধোয়া ধুয়ির পানি Washing water…………………………<br>রান্নার তেলCooking oil…………………………………<br>চা/কফি Tea/Coffee…………………………………….<br>ডাল/অন্য তরল খাবারSoup/other liquid food…………….<br>ভাতের মাড় Rice water………………………………….<br>রাইস মিলের গরম পানি Rice mill boiler source……………<br>বাড়ীতে ধান সিদ্ধের গরম পানি………………………………<br>শিল্প কারখানার তরল Liquid in a factory………………….<br>অন্যান্য (উল্লেখ করুন) Others (Specify) ________________	01<br>02<br>03<br>04<br>05<br>06<br>07<br>08<br>09<br>10<br>97	} L07
L 04.	যদি উত্তপ্ত বস্তু হয়, বস্তুটি কি ছিল ?	রান্নার তৈজষপত্র Cooking utensils…………………………<br>কয়লা Coal……………………………………………..<br>ইস্ত্রী Iron…………………………………………….....<br>ইঞ্জিনের অংশ Muffler/engine part…………………….<br>হিটার Heater…………………………………………….<br>কর্মস্থলের উত্তপ্ত বস্তু Workplace source……………………<br>অন্যান্য (উল্লেখ করুন) Others (Specify) ________________	01<br>02<br>03<br>04<br>05<br>06<br>97	} L07

L 05.	যদি বিস্ফোরক হয়, কেন তা ব্যবহার করা হয়েছিল ?	আতশবাজিFire cracker/fire work……………………………..<br>নির্মাণ কাজে Construction…………………………………<br>সন্ত্রাসী কার্যকলাপে Terrorism………………........................<br>অন্যান্য (উল্লেখ করুন) Others (Specify) ______________	01<br>02<br>03<br>97	L07
L 06.	যদি রাসায়নিক পদার্থে পুড়ে থাকে, তবে পদার্থটি কি ?	এসিড Acid…………………………………………..….<br>চুন Lime……………………………………………………..<br>অন্যান্য (উল্লেখ করুন) Others (Specify) _______________	01<br>02<br>97	
L 07.	সে সময় পুড়ে যাওয়া ব্যক্তিটি কি করছিল ?	কর্মস্থলে কাজে ছিল During work (outside home)…………<br>বাড়ীতে রান্না করছিল House work (cooking)….……………<br>রান্নাছাড়াঅন্যান্য গৃহস্থালীরকাজ…………………………<br>বাড়িতে ধান সিদ্ধ করছিল …………………………………..<br>অন্যান্য (উল্লেখ করুন) Others (Specify)_____________ ___	01<br>02<br>03<br>04<br>97	END

Module V (M7) Section M: এম ৭- পানিতে ডুবা

House hold identification number: ☐ ☐☐☐☐☐☐☐☐

Person Number: ☐☐

Person Name:

No.	Questions	Coding Categories		Skip
M01.	ব্যক্তিটি (নাম উল্লেখ করুন) কোথায়ডুবেগিয়েছিল?	**কক্ষের মধ্যে - Indoor**<br>বালতি Bucket……………………………………….……<br>ড্রাম Drum…………………………………………...<br>চারি Tub………………………………………….....<br>পানির ট্যাঙ্ক/হাউজ Household water reserv…………<br>**কক্ষের বাইরে - Outdoor**<br>পুকুর Pond………………………………………….....<br>ডোবা Ditch…………………………………….…<br>কুয়া (কূপ) Well………………………………..……<br>চারি Tub………………………………………........<br>খাল ………………………………………..………..<br>নদী …………<br>সমুদ্র Sea………………………………………………<br>পানির ট্যাঙ্ক/হাউজ Water reservoir…………………………<br>অন্যান্য (উল্লেখ করুন) Others (Specify)_____________ ____________	01<br>02<br>03<br>04<br><br>05<br>06<br>07<br>08<br>09<br>10<br>11<br>12<br>97	
M02.	কক্ষের মধ্যে হলে জলাধারটি কোথায় ছিল ?	গোসলখানা/পায়খানা Bathroom……………………………………..<br>রান্নাঘরে Kitchen…………………………………………….............<br>এক কক্ষ বিশিষ্ট ঘরে Single room dwelling………………………………<br>বাড়ীর ছাদে Roof……………………………………………………….<br>মাটির নীচেUnderground water reservoir……………………………<br>অন্যান্য উল্লেখ করুন Others (Specify ________________________	01<br>02<br>03<br>04<br>05<br>97	
M03.	কক্ষের বাইরে হলে বাড়ী থেকে জলাশয়ের দূরত্ব কত?	__________________		
M04.	জলাধারটি কি ঢাকা ছিল ?	ঢাকা ছিল না Not covered………………………<br>আংশিক ঢাকা ছিল Partially covered………………………….<br>সম্পূর্ন ঢাকা ছিল Covered……………………...……<br>প্রযোজ্য নয় (নদী, সমুদ্র,পুকুর ইত্যাদি) …………………………	01<br>02<br>03<br>88	
M05.	জলাশয়টি কি কাজেব্যবহার হয়?	গোসল ও ধোয়াধুয়িকরা Bathing and washing……………………………..<br>রান্না ও পানকরারপানিসংগ্রহকরা Cooking and drinking……………………..<br>মাছ চাষ Farming fish……………………………………………….<br>তেমন কোনব্যবহার নেই No regular household use……........................<br>অন্যান্য (উল্লেখ করুন) Others (Specify)………………………………	01<br>02<br>03<br>04<br>97	
M06.	যদি বাড়ির বাইরে হয় তবে জলাশয়টির চারি দিক কি ঘেরাছিল?	হ্যাঁYes……………………………………....<br>নাNo………………………………………….<br>জানা নেই Don’t know………………..............	01<br>02<br>99	

M07.	ডুবে যাওয়ার আগে ব্যক্তিটি কি করছিল ?	জলাশয়ে খেলা করছিল্‌……………………………………………<br>জলাশয়ে গোসল / ওযু/ সাঁতার কাটছিল ………………………………<br>ধোয়াধুয়িকরছিল Washing…………………………….…………..<br>জলাশয়েরধারেকাজ/খেলাকরছিল Work/play near the reservoir…………<br>পানিআনতেগিয়েছিল Fetching water…………………………………<br>জলাধারের পাড় দিয়ে হাঁটার সময় পা পিছলে পড়েগিয়েছিল ……………<br>মাছ ধরছিল Fishing………………………………………………….<br>যাত্রী (নৌকা/ফেরী/লঞ্চ) Travelling onboat / ferry / launch………..…<br>অন্যান্য (উল্লেখ করুন) Others (Specify) _____________ _________<br>জানা নেই Don't know………………………………………………..	01<br>02<br>03<br>04<br>05<br>06<br>07<br>08<br>97<br>99	
M08.	ব্যক্তিটি কি সাঁতার কাটতে জানতো ?	হ্যাঁ Yes……………………………………………………………<br>না No……………………………………………………………….<br>প্রযোজ্য নয় (যদি শিশুর বয়স ৪ বছরের কম হয়) . ……………………<br>জানা নেই Don't know………………………………………………..	01<br>02<br>88<br>99	
M09.	ঘটনার পূর্ব মুহূর্তে তার সঙ্গে কে ছিল ?	মা Mother…………………………………………………………..<br>বাবা Father………………………………………………………...<br>পরিচর্যাকারী Caregiver……………………………………………...<br>বড়ভাই/বোন Elder sibling…………………………………………<br>স্বামী/স্ত্রী Spouse…………………………………………………<br>অন্য আত্নীয় স্বজন Other relative……………………………………<br>বন্ধু /সমবয়সীশিশু Friend/Peer……………………………………<br>সহকর্মী Colleague…………………………………………………<br>কেউ নয় No one……………………………………………………....<br>অন্যান্য (উল্লেখ করুন) Others (Specify)________________________<br>জানা নেই Don't know……………………………………………	01<br>02<br>03<br>04<br>05<br>06<br>07<br>08<br>09<br>97<br>99	 <br><br><br><br><br><br><br><br>→M11<br>→M11
M10.	সঙ্গীর বয়স কত ছিল ? (বছরে)	|__|__|		
M11.	সে সময়মা / পরিচর্যাকারীকিকরছিলেন ?	গৃহকর্ম করছিলেনDoing household chores…………………………<br>অন্যদের সঙ্গে গল্পকরছিলেন Chatting with others……………….................<br>ঘুমাচ্ছিলেনSleeping…………………………………………………<br>বাড়ীরবাইরেকাজেছিলেনWorking outside home………………….............<br>শিশুটির সাথে ছিলেনAccompanying the child…………………………….<br>খাচ্ছিলেনHaving meal……………………………………………..<br>অন্য শিশুরপরিচর্যাকরছিলেনLooking after another child………………….<br>প্রতিবেশীরবাড়ী বেড়াতেগিয়েছিলেনVisiting neighbor ……………………<br>অন্যান্য (উল্লেখ করুন) Others (Specify)_____________ __________	01<br>02<br>03<br>04<br>05<br>06<br>07<br>08<br>97	
M12.	কেউ কি তাকে পানি থেকে উদ্ধার করেছিল?	হ্যাঁ Yes……………………………………………………………<br>না No……………………………………………………………….<br>জানা নেই Don't know……………………………………………	01<br>02<br>99	 →M14<br>→M14

M13.	কে তাকে উদ্ধার করেছিল?	মা Mother……………………………………………………………..<br>বাবা Father…………………………………………….……….<br>প্রাপ্ত বয়স্ক পরিচর্যাকারী Other adult caregiver………………………<br>বড় ভাই বোন Elder sibling……………………………………………..<br>বন্ধু / সমবয়সী শিশু Friend/peer………………………………………<br>প্রতিবেশী Neighbor…………………………………………………......<br>অন্যান্য (উল্লেখ করুন) Others (Specify)_________________________<br>জানা নেই Don't know…………………………………………………	01<br>02<br>03<br>04<br>05<br>06<br>97<br>99	
M 14	পানিতে ডুবা থেকে উদ্ধার করার পর তাকে কি করা হয়েছিল ?	মাথায় তুলে ঘুরানো হয়েছিল …………………………………………………………<br>পেটে চাপ দিয়ে বমি করানো হয়েছিল …………………………………………………..<br>শরীরে তেল মালিশ করা হয়েছিল ……………………………………………………..<br>মুখে ছাই দেওয়া হয়েছিল …………………………………………………………<br>মুখে শ্বাস ও বুকে চাপ দেওয়া হয়েছিল ………………………………………………<br>দ্রুত ডাক্তারের নিকট নেওয়া হয়েছিল …………………………………………………..	01<br>02<br>03<br>04<br>05<br>06	
M14.	আপনার মতে, কি করলে শিশুদের পানিতে ডুবে যাওয়া থেকে রক্ষা করা যায় ?	সার্বক্ষনিক দেখাশুনা করা Constant supervision…………………………….<br>সার্বক্ষনিক খেয়াল রাখার বিশেষ ব্যবস্থা করা (যেমনপায়ে/কোমরে ঘুঁঙুরবাঁধা, ইত্যাদি)…<br>শিশুটিকে পানিতে যেতেনা দেওয়া (পুকুর ঘেরা, বেবী কট, প্লেপেন)........................<br>অব্যবহার্য / অপ্রয়োজনীয়জলাশয়ভরাটকরা..<br>সাঁতার শেখানো Teaching swimming ………………………………<br>পানি থেকে উদ্ধারকরার সাথে সাথে সঠিক প্রাথমিক চিকিৎসা ও পরবর্তী ব্যবস্থাপনা -----<br>পানিতেডুবে মৃত্যুর বিষয়ে জনসচেতনতা বৃদ্ধি করা…………………………….<br>কোন ধারনা নেই No idea……………………………………………<br>অন্যান্য (উল্লেখ করুন) Others (Specify) ________________________	01<br>02<br>03<br>04<br>05<br>06<br>07<br>08<br>97	END

Module V (M8) Section N: এম ৮-দুর্ঘটনাজনিত বিষক্রিয়া

House hold identification number: ☐ ☐☐☐☐☐☐☐☐

Person Number: ☐☐

Person Name:

No.	Questions	Coding Categories		Skip
N 01.	বিষের ধরণ কি ছিল ?<br>What was the type of poison?	কীটনাশক Pesticides………………………………………..<br>মশা, শাছি, তেলাপোকাইত্যাদি ধ্বংসকারী Insecticides...................<br>ইঁদুর মারা বিষ Rodenticides……………………………...<br>ঘুমের বড়ি Sleeping pills……………................................<br>অন্যান্য ঔষধ Other medicine………………………………..<br>সাবান/গুঁড়া সাবান Soap/detergent………..…....................<br>কেরোসিন Kerosene…………..…………………………<br>স্যাভলন/ডেটল Savlon/Dettol…………………………………<br>Harpic<br>অন্যান্য (উল্লেখ করুন) Others (Specify)..............................<br>জানা নেই Don't know………………………..……........	01<br>02<br>03<br>04<br>05<br>06<br>07<br>08<br>09<br>10<br>97<br>99	
N 02.	কি ধরনের পাত্রে বিষ রাখা হয়েছিল ?<br>In what type of container was the poison kept?	বোতল Bottle………………………………………………......<br>কৌটা Container ………………………………………<br>প্যাকেট Packet…………………………………………<br>ষ্ট্রিপ Strip………………………………………………..<br>অন্যান্য (উল্লেখ করুন) Others (Specify)……………………	01<br>02<br>03<br>04<br>97	
N 03.	পাত্রটি কি প্রকৃত না অন্য ছিল ?<br>Was the container original?	বিষের নিজস্ব বোতল (প্রকৃত) Original………………………<br>অন্য বোতলে ছিল (পেপসি, কোকাকোলা, মজো) ………………..	01<br>02	→N05
N 04.	যদি অন্য পাত্র হয় তবে কি সেটা লেবেল করা ছিল ?<br>If not original, was it labeled?	লেবেল করা Labeled………………………………………<br>লেবেল বিহীন Not labeled…………………………………	01<br>02	
N 05.	যদি বোতল হয় তবে পাত্রের মুখ বন্ধ ছিল কি ?<br>If bottle, was the container capped?	হ্যাঁ Yes…………………………………………………<br>না No……………………………………………………..<br>জানা নেই Don't know……………………………………	01<br>02<br>99	
N 06.	পাত্রটি কোথায় রাখা হয়েছিল ? (স্থান)<br>Where was contain the pack (placed)?	মেঝেতে Floor……………………………………........<br>খাট/চৌকির নীচেUnder bed……………………………..<br>তোষক/ পাটির নিচেUnder mattress……………………..........<br>বাক্সের মধ্যে Inside box……………………………….........<br>সেল্ফ এর উপরে (১ মিটারের উর্দ্ধে)…………….................<br>সেল্ফ এর উপরে (১ মিটারের নীচে)…………………………<br>সিলিং এ ঝুলন্ত অবস্থায় Hanging from ceiling……………<br>মাচায় ঝুলন্ত অবস্থায় Bamboo shelf (*macha*)……………….<br>অন্যান্য (উল্লেখ করুন) Others (Specify)……………………	01<br>02<br>03<br>04<br>05<br>06<br>07<br>08<br>97	
N 07.	পাত্রটি কি অবস্থায় ছিল ?	খোলা অবস্থায় Open place…………………………………...<br>তালাবদ্ধ অবস্থায় Locked…………………………………….	01<br>02	
N 08	**ঘটনা ঘটার/বিষক্রিয়ার স্থান কোথায় ছিল**	ঘরের ভিতর ……………………………………………..……<br>উঠানে ……………………………………………..……<br>শস্য ক্ষেতে ……………………………………..……<br>জানা নেই ……………………………………………..……	01<br>02<br>03<br>99	
N 09	a) **শিশুর বিষক্রিয়ার মাধ্যম**	মুখে দেয়ার মাধ্যমে…………………………………………..……<br>খাবারের মাধ্যমে…………………………………………..……<br>শ্বাসপ্রশ্বাসের মাধ্যমে…………………………………………..……<br>ত্বকের অভ্যন্তরে কোনভাবে প্রবেশের মাধ্যমেত্বকের সংস্পর্শের মাধ্যমে…<br>অন্যান্য(উল্লেখ করুন) Others (Specify)……………………	01<br>02<br>03<br>04<br>97	

Module V (M9) Section O: এম ৯- মেশিন/যন্ত্রপাতির দ্বারা জখম

House hold identification number: ☐ ☐☐☐☐☐☐☐☐

Person Number: ☐☐

Person Name:

No.	Questions	Coding Categories		Skip
O 01.	এটা কি ধরনের মেশিন ছিল ?	কৃষি কাজে ব্যবহৃত মেশিন Agricultural…………………....	01	
		শিল্প/কারখানায় ব্যবহৃত মেশিন Industrial……………………	02	→ O03
		নির্মাণ কাজে ব্যবহৃত মেশিন Construction………………	03	→ O04
		যানবাহনের ইঞ্জিন/ মেশিন ………………………………	04	→ O05
		অন্যান্য (উল্লেখ করুন) Others (Specify)……………………	97	→ End
O 02.	কৃষি মেশিন হলে, কি ধরনের মেশিনছিল ?	শ্যালো মেশিন ও অন্যান্য পাম্প Shallow machine or other pumps ..	01	End
		ট্রাকটর Tractor……………………………………………	02	
		ধান মাড়াই এর কল Rice grain separating machine…………..	03	
		অন্যান্য যন্ত্রচালিত মেশিন Other powered machine…………	04	
O 03.	শিল্প কারখানার মেশিন হলে, এটা কি ধরনের মেশিন ছিল?	রাইস মিল Rice milling/polishing machine…………………	01	End
		প্রেস/ছাপখানা Pressing machine……………………….	02	
		ছিদ্র করার যন্ত্র/ড্রিল মেশিন Drilling machine…………………..	03	
		করাত কল Sawing machine…………………………………	04	
		ওয়েল্ডিং মেশিন Welding machine…………………………	05	
		ধাতব্য বস্তু কাটার যন্ত্র Metal cutting machine ……………	06	
		পিশন যন্ত্র Grinding machine………………………......	07	
		সেলাই মেশিন Sewing machine…………………………	08	
		তাঁত যন্ত্র Power loom ……………………………………..	09	
		অন্যান্য (উল্লেখ করুন) Others (Specify)……………………	97	
O 04.	এটা কি ধরনের নির্মাণ কাজে ব্যবহৃত মেশিন ছিল?	মিক্সচার মেশিন Mixture machine…………………….……	01	End
		ভাইব্রেটর Vibrator…………………………………….……	02	
		মোজাইক/ টাইলস কাটার Mosaic cutter machine ……………..	03	
		নির্মান সামগ্রী উপরে তোলার যন্ত্র Pulling machine ……………	04	
		মাটি খনন যন্ত্র ……………..……………….....…...…	05	
		অন্যান্য (উল্লেখ করুন) Others (Specify)………………...…	97	
O 05	যানবাহনের ইঞ্জিন	যানবাহনের ইঞ্জিন ………....………………...…...…	01	
		নসিমন/ ভটভটির ইঞ্জিন………....………………...…...…	02	
O 06	ইনজুরি ঘটার সময় ব্যক্তিটি কি করছিল ?	মেশিনটি চালু করছিল ……....………………...…...…	01	
		মেশিন দিয়ে কাজ করছিল ……....………………...…...…	02	
		মেশিনের কাছে অবস্থান করছিল ……....………………...…...…	03	
		অন্যান্য (উল্লেখ করুন) Others (Specify)………………...…	97	

Module V (M10) Section P: এম ১০-বিদ্যুতস্পৃস্টের তথ্য

House hold identification number: ☐☐ ☐☐☐☐☐☐☐

Person Number: ☐☐

Person Name:

No.	Questions	Coding Categories		Skip
P 01.	বিদ্যুতের উৎস কি ছিল ?	বজ্রপাত Lightning……………………………………..…	01	
		বাড়ীতে ব্যবহৃত বিদ্যুৎ Electricity inside home…………..…	02	
		বাড়ীর বাহিরে ব্যবহৃত বিদ্যুৎ Electricity outside of home……	03	→ P03
		কারখানায় ব্যবহৃত বিদ্যুৎ Electricity used in a factory ……..	04	→ P03
		বাড়ি বা কারখানা ব্যতীত বিল্ডিংএ ব্যবহৃত বিদ্যুৎ……………	05	→ P03
P 02.	যদি বাড়ীতে হয়, কোথায় ঘটেছিল ?	রান্না ঘর Kitchen……………………………………...…	01	
		বসার ঘর Living area …………………………………....	02	
		শোয়ার ঘর Bedroom………………………………………	03	
		এক কক্ষ বিশিষ্ট ঘর Single room dwelling ……………..……	04	
		গোসলখানা/পায়খানা Bathroom…………………………..	05	
		বারান্দা Veranda……………………………………………..	06	
		অন্যান্য (উল্লেখ করুন) Others (Specify) ______________	97	
P 03.	যদি বাড়ীর বাহিরে হয়, কোথায় বিদ্যুৎস্পৃষ্ট হয়েছিল?	রাস্তায় Street…………………………………………….	01	
		মাঠে Field ………………………………………………..	02	
		অফিসে Office……………………………………………	03	
		শিল্প প্রতিষ্ঠানে Factory/Industry………………………	04	
		হাট/বাজার Market………………………………………	05	
		জলাশয়ে…………………………………………………	06	
		গাছ তলায়…………………………………………………	07	
		অন্যান্য (উল্লেখ করুন) Others (Specify)______________	97	
P 04	কিভাবে বিদ্যুতায়িত হয়েছিল ?	বিদ্যুতের তার/ সুইচে ছোঁয়ার মাধ্যমে………………………………	01	END
		বিদ্যুতের তার ছিঁড়ে পড়ে……………………………………	02	
		বজ্রপাতের মাধ্যমে……………………………………	03	

Module V (M11) Section T: এম ১১- প্রাণী/কীটপতঙ্গের দ্বারা জখম

House hold identification number:	☐ ☐☐☐☐☐☐☐☐
Person Number:	☐☐
Person Name:	

No.	Questions	Coding Categories		Skip
T 01.	প্রাণী/ কীটপতঙ্গটি কি ছিল ?	পোষা কুকুর Pet dog……………………………………	01	
		রাস্তার কুকুর Stray dog……………………………………	02	
		পোষা বিড়াল Pet cat………………………………………	03	
		রাস্তার বিড়াল Stray cat ……………………..……………..…	04	
		সাপ Snake…………………………………………….….…	05	
		শিয়াল Fox……………………………………………….……	06	
		গরু Cow……………………………………………………...	07	
		মহিষ Buffalo… ……………………………….…….	08	
		ভিমরুলHornet……………………………………………….	09	
		বোলতা Wasp…………………………………………….……	10	
		মৌমাছি Bee……………………………………………..	11	
		অন্যান্য (উল্লেখ করুন) Others (Specify)_________________	97	
T 02.	এটা কি ধরনের ছিল ?	কামড়/আঁচড় Bite……………………………………...	01	
		হুলফুটানো Sting……………………............................	02	
		লাথি Kick………………………...................................	03	
		পায়েরতলায়পড়া Step on………………………..................	04	
		শিংবামাথাদিয়েআঘাত Horn/other butting/impale…...............	05	
		অন্যান্য (উল্লেখ করুন) Other (specify)..............................	97	
T 03.	আক্রমণের পূর্ব মুহুর্তে ব্যক্তিটি কি করছিল ?	খেলা / বিরক্ত করার কারনে Playing /disturbing……………..….	01	
		খাওয়ানো হচ্ছিল Feeding………………..............................	02	
		কাজে ব্যবহার করা হচ্ছিল Using for work…………………	03	
		বিরক্ত করা ছাড়াই Unprovoked, no prior involvement………	04	
		অন্যান্য (উল্লেখ করুন) Others (Specify) ________________	97 →	END

Module V (M12) Section Q: এম ১২- ভোঁতা বস্তু দ্বারা আঘাত

House hold identification number:				
Person Number:				
Person Name:				

No.	Questions	Coding Categories		Skip
Q 01.	ভোঁতা বস্তুটি কি চলন্ত /স্থির ছিল ?<br>Was the blunt object moving or fixed?	চলন্ত বস্তু (নাম লিখুন) Moving objects (specify)……………..<br>স্থির বস্তু (নাম লিখুন) Fixed objects (specify)…………......<br>অন্যান্য (উল্লেখ করুন) Others (Specify)________________	01<br>02<br>97	
Q 02.	ভোঁতা বস্তুটি কি কাজে ব্যবহার করা হচ্ছিল ?<br>What was the blunt object used for?	চাষাবাদ/খামারের কাজে Farm……………………………….<br>গৃহস্থালীর কাজে Household………………………………..<br>কল-কারখানার কাজে Factory………………………………<br>দোকানের কাজে Shop………………………………………<br>নির্মাণ কাজে Construction……………………………….<br>অফিস আদালতে Office………………………………………<br>খেলাধূলায় Game………..<br>অন্যান্য (উল্লেখ করুন) Others (Specify)_____________	01<br>02<br>03<br>04<br>05<br>06<br>07<br>97	END<br>→

Blunt object like:

লাঠি, হাতুড়ি, খেলার ব্যাট-এর কোন ভোঁতা প্রান্ত দ্বারা আহত হওয়া , দরজা বা জানালায় চাপা পড়ে আহত হওয়া

stick, hammer, injured from sporting bat with blunt edges, bruised skin in between doors or windows

Module V (M13) Section R: এম ১৩- শ্বাসরোধ

House hold identification number: ☐ ☐☐☐☐☐☐☐☐

Person Number: ☐☐

Person Name:

No.	Questions	Coding Categories		Skip
R 01.	**শ্বাসরোধের কারণ?**	কার্বন মনোক্সাইড Carbon monoxide……………….…...	01	
		অন্য গ্যাস (উল্লেখ করুন) Other gas (specify)………………	02	
		তরল(উল্লেখ করুন) Liquid (specify)…………….....................	03	
		মাছের কাঁটা Fishbone…………………………………...	04	
		অন্য খাদ্যবস্তু(উল্লেখ করুন) Other food items (specify)……	05	
		ধাতব মুদ্রা (পয়সা/টাকা) Coin……………………………	06	
		ছোট বস্তু (উল্লেখ করুন) Other Small object (specify)……	07	
		কাপড়ে ঢেকে যাওয়ার কারণে Covered by clothes…………	08	
		প্লাস্টিকেরব্যাগ/দ্রব্য দ্বারাঢেকে যাওয়া………………………………	09	
		বড় ব্যক্তির শরীরের কোন অংশের চাপে Covered by adult body…		
		মাটি চাপা পড়ায় Covered by earth………………………	10	
		পানির ট্যাংক / সেফটি ট্যাংক বা কুয়াতে নামার ফলে ………………	11	
		অন্যান্য (উল্লেখ করুন) Others (Specify) _____________	12	
			97	
R 02.	**ঘটনার সংক্ষিপ্ত (সর্বোচ্চ তিনটি বাক্যে) বিবরন**	…………………………………………………………………<br>…………………………………………………………………<br>…………………………………………………………………<br>…………………………………………………………………<br>………………………………………………………….………		END

Module VI

Section S: Quality of life assessment

Special Note: By placing a tick in one box in each group below, please indicate the statements that best describe your own health state today.

No.	Issues	Indicators	
S1	Mobility	I have no problems in walking	☐
		I have some problems in walking	☐
		I am confined to bed	☐
S2	Self-Care	I have no problems with self-care	☐
		I have some problems bathing or dressing myself	☐
		I am unable to bathe or dress myself	☐
S3	Usual Activities*(e.g. work, study, household work, family orleisure activities)* (, , ,)	I have no problems in performing my usual activities	☐
		I have some problems in performing my usual activities	☐
		I am unable to perform my usual activities	☐
S4	Pain / Discomfort	I have no pain or discomfort	☐
		I have moderate pain or discomfort	☐
		I have extreme pain or discomfort	☐
S5	Anxiety / Depression	I am not anxious or depressed	☐
		I am moderately anxious or depressed	☐
		I am extremely anxious or depressed	☐

To help people say how good or bad a health state is, we have drawn a scale (somewhat like a thermometer) on which the best state you can imagine is marked 100 and the worst state you can imagine is marked 0.

We would like you to indicate on this scale how good or bad your own health is today, in your opinion. Please do this by drawing a line from the box below to whichever point on the scale indicates how good or bad your health state is today.

(

)

Your ownhealthstatetod ay

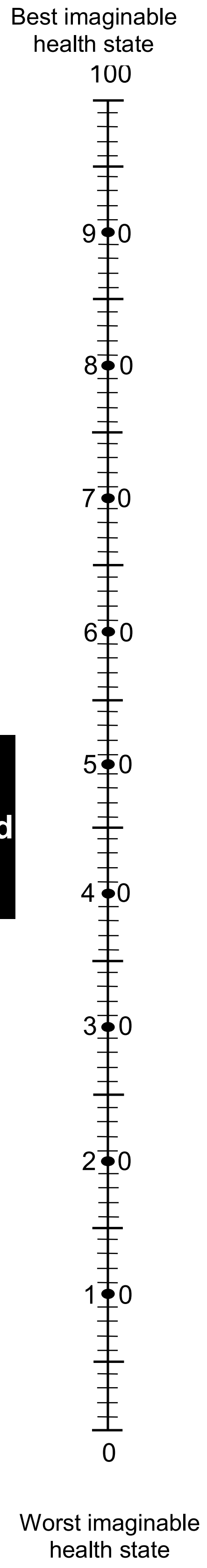

Supplement: S1 File — The questionnaire used in the Bangladesh Health and Injury Survey (BHIS), 2016. (PDF) [file pone.0353183.s001.pdf]
