# Supplementary material for: Epidemiology and healthcare burden of non-fatal maxillofacial injuries in Bangladesh: Evidence from Bangladesh Health and Injury Survey (BHIS), 2016
Source: PLoS One. 2026 Jul 9;21(7):e0353183. doi: 10.1371/journal.pone.0353183 (PMC13349135; doi:10.1371/journal.pone.0353183)
Supplement: S2 File — (DOCX) [file pone.0353183.s002.docx]

**S2 File. Determination of the sample size**

In BHIS 2003, the injury mortality rate was 50 per 100,000 population per year. For BHIS 2016, assuming the same injury mortality (50 per 100,000; p=0.0005), a 95% confidence interval, and 15% precision (d=0.15×p=0.000075), the required sample size was calculated as 341,163. Although a design effect of 2 (because of the two-stage cluster sampling) would have doubled the required sample, a two-year recall period (for injury mortality) was adopted for logistical reasons, and the calculated sample size was therefore retained at 341,163. Allowing for a 2.5% non-response rate produced a final calculated sample size of 361,690. Consequently, a rounded sample of approximately 350,000 individuals was targeted for coverage in the entire survey.

The following formula was used to calculate the sample size:

$$n = \frac{Z^{2}ₐ/₂ . p. (1-p)}{d^{2}}$$

Where, Z= 1.96, p = 0.0005, (1-p) = 0.9995, and d (precision level) = 0.000075
